# Introducing the antibacterial and photocatalytic degradation potentials of biosynthesized chitosan, chitosan–ZnO, and chitosan–ZnO/PVP nanoparticles

**DOI:** 10.1038/s41598-024-65579-z

**Published:** 2024-06-26

**Authors:** Abdelatif Aouadi, Djamila Hamada Saud, Abdelkrim Rebiai, Abdelhak Achouri, Soulef Benabdesselam, Fatma Mohamed Abd El-Mordy, Pawel Pohl, Sheikh F. Ahmad, Sabry M. Attia, Hamada S. Abulkhair, Abderrahmane Ararem, Mohammed Messaoudi

**Affiliations:** 1 Process Engineering Laboratory, Applied Sciences Faculty, Kasdi Merbah University, 30000 Ouargla, Algeria; 2 Laboratory of Applied Chemistry and Environment, Faculty of Exact Sciences, University of Hamma Lakhdar El-Oued, B.P.789, 39000 El-Oued, Algeria; 3 Water, Environment and Sustainable Development Laboratory (2E2D), Faculty of Technology, University of Blida 1, Route Soumâa, BP 270, Blida, Algeria; 4 Laboratory of Water and Environmental Engineering in the Saharan Environment, Process Engineering Department, Faculty of Applied Sciences, Kasdi Merbah-Ouargla University, Ouargla, Algeria; 5 Department of Pharmacognosy and Medicinal Plants, Faculty of Pharmacy (Girls), Al-Azhar University, Cairo, 11754 Egypt; 6 Department of Analytical Chemistry and Chemical Metallurgy, Faculty of Chemistry, University of Science and Technology, Wyspianskiego 27, 50-370 Wrocław, Poland; 7 Department of Pharmacology and Toxicology, College of Pharmacy, King Saud University, 11451 Riyadh, Saudi Arabia; 8 Pharmaceutical Organic Chemistry Department, Faculty of Pharmacy, Al-Azhar University, Nasr City, Cairo, 11884 Egypt; 9 Pharmaceutical Chemistry Department, Faculty of Pharmacy, Horus University-Egypt, International Coastal Road, New Damietta, 34518 Egypt; 10 Nuclear Research Centre of Birine, P.O. Box 180, 17200 Ain Oussera, Djelfa Algeria

**Keywords:** Zinc oxide, Chitosan, Gram-positive bacteria, Anti-bacterial agents, ProTox-II, Biochemistry, Biotechnology, Drug discovery, Biogeochemistry, Environmental social sciences

## Abstract

The development of nanomaterials has been speedily established in recent years, yet nanoparticles synthesized by traditional methods suffer unacceptable toxicity and the sustainability of the procedure for synthesizing such nanoparticles is inadequate. Consequently, green biosynthesis, which employs biopolymers, is gaining attraction as an environmentally sound alternative to less sustainable approaches. Chitosan-encapsulated nanoparticles exhibit exceptional antibacterial properties, offering a wide range of uses. Chitosan, obtained from shrimp shells, aided in the environmentally friendly synthesis of high-purity zinc oxide nanoparticles (ZnO NPs) with desirable features such as the extraction yield (41%), the deacetylation (88%), and the crystallinity index (74.54%). The particle size of ZnO NPs was 12 nm, while that of chitosan–ZnO NPs was 21 nm, and the bandgap energies of these nanomaterials were 3.98 and 3.48, respectively. The strong antibacterial action was demonstrated by ZnO NPs, chitosan–ZnO NPs, and chitosan–ZnO/PVP, particularly against Gram-positive bacteria, making them appropriate for therapeutic use. The photocatalytic degradation abilities were also assessed for all nanoparticles. At a concentration of 6 × 10^–5^ M, chitosan removed 90.5% of the methylene blue (MB) dye, ZnO NPs removed 97.4%, chitosan-coated ZnO NPs removed 99.6%, while chitosan–ZnO/PVP removed 100%. In the case of toluidine blue (TB), at a concentration of 4 × 10^–3^ M, the respective efficiencies were 96.8%, 96.8%, 99.5%, and 100%, respectively. Evaluation of radical scavenger activity revealed increased scavenging of ABTS and DPPH radicals by chitosan–ZnO/PVP compared to individual zinc oxide or chitosan–ZnO, where the IC50 results were 0.059, 0.092, 0.079 mg/mL, respectively, in the ABTS test, and 0.095, 0.083, 0.061, and 0.064 mg/mL in the DPPH test, respectively. Moreover, in silico toxicity studies were conducted to predict the organ-specific toxicity through ProTox II software. The obtained results suggest the probable safety and the absence of organ-specific toxicity with all the tested samples.

## Introduction

The evolution of materials science via nanotechnology has brought zinc oxide nanoparticles (ZnO NPs), which have extraordinary properties in optics, electronics, photocatalysis, and antiseptics. Due to their biodegradability and low toxicity, these NPs find applications in a variety of industries, including healthcare and environmental cleanup, making them good candidates for drug delivery systems^1,2^. With a binding energy of 60 MeV and an energy band of 3.37 eV, ZnO NPs stability can be modified for particular applications by altering their shape through synthetic procedures and modifiers. Recent research aimed to improve the photocatalytic performance of ZnO NPs by lowering the bandgap energy through their doping with some metals or nonmetals^3,4^. ZnO NPs demonstrated a strong antibacterial activity, and hence, they were established as very promising food preservatives against the action of key foodborne pathogens such as *Listeria monocytogenes*, *E. coli* O157:H7, *Campylobacter jejuni,* and *Salmonella* spp. Importantly, ZnO NPs received regulatory approval from the Food and Drug Administration (FDA), indicating that they are safe for use in a variety of applications^5^. These NPs are particularly attractive because they could be used as broad-spectrum antibiotics to overcome microbial resistance against existing antibiotics^6^. Their action on microorganisms is based on the production of the reactive oxygen species, the attachment to the microbial cells, and the entrance to the cells, among other methods. Although it is possible, in this case, the development of resistance by the bacteria is less frequent than in conventional antibiotics^7^.

The use of biopolymer molecules or plant extracts for the green biosynthesis of NPs is growing in popularity since the physicochemical-based synthesis is criticized for its impact on the environment and due to high costs^8,9^. Chitosan, an aminated polysaccharide derived from crustaceans and insects, is a promising material for the synthesis of metal and metal oxide NPs, particularly ZnO NPs. Its amino groups improve the potential of metallic NPs to remove a range of pollutants including heavy metals, harmful pesticides, and dyes^10^. The amino and hydroxyl functionalities of chitosan act also to enhance the biocompatibility of the metallic NPs^11^. The NPs with the chitosan coating are recognized to exhibit antibacterial effects against the *Candida* species and other bacterial and fungal microorganisms^12^. The ZnO NPs are of great importance in the case of the photocatalytic destruction of organic pigments, offering an efficient and eco-friendly way to couple with water contamination^12^.

The antibacterial activity of both chitosan and ZnO NPs has been individually investigated before^13,14^. For instance, ZnO nanoparticles on one hand were recently synthesized and showed noteworthy photocatalytic activity in degrading methylene blue^15^. More recently, polyethyleneglycol-coated ZnO NPs were hydrothermally developed, and their potential antibacterial activity, presented by MIC and MBC, was evaluated against a set of Gram-negative and Gram-positive strains. ZnO NPs showed a bacteriostatic effect on *E. coli* and *P. aeruginosa. They also revealed* a bactericidal effect on *S. aureus* and *K. pneumoniae*^16^. On the other hand, the photocatalytic and antibacterial activities of chitosan nanomaterials on methylene blue were also reported^11^. Additionally, polymeric blends of PVP with silver and ZnO nanoparticles displayed photocatalytic and antimicrobial potentials^17–19^. The main objectives of this work are to investigate the antibacterial properties of chitosan-coated ZnO NPs as well as to conduct a comparative analysis between chitosan extracted from shrimp peels and ZnO NPs synthesized with the aid of chitosan as a reducing agent. Additionally, the potential of chitosan-coated ZnO NPs in removing organic colorants from water through photocatalysis has been investigated. Through the comprehensive examination of the properties, the performance, and the environmental sustainability of both ZnO NPs and chitosan-coated ZnO NPs, this study aimed to enhance the understanding of these materials and their potential suitability for several industrial applications. By highlighting the unique features and potential benefits of chitosan and chitosan-coated ZnO NPs, the present work contributes to the advancement of sustainable materials research and promotes their practical utilization in various fields.

## Materials and methods

### Materials

#### Plant materials

*Solanum nigrum* L. leaves were collected in the summer of 2022 from eastern Algeria; specifically, from El-Oued region. *Solanum nigrum* L. was identified by Pr. Chehma A. (Ouargla University) and a voucher specimen (SN-S22-09) was deposited in the Laboratory of Process Engineering, Faculty of Applied Sciences, Kasdi Merbah University, Ouargla, 30000, Algeria. The collection of *Solanum nigrum* L. leaves samples was carried out after obtaining permission from local suppliers. The authors confirm that this study complies with relevant legislation and international, national, and institutional guidelines.

#### Chemicals

Zinc chloride (ZnCl_2_, 99%), sodium hydroxide (NaOH, 97%), hydrochloric acid (HCl, 99%), acetic acid (CH_3_COOH, 99.5%), methylene blue (C_16_H_18_ClN_3_S, 82%), hydrogen peroxide (H_2_O_2_, 98%), and dimethyl sulfoxide (DMSO, 99%) were purchased from Biochem-Chemo pharma. Mueller–Hinton agar was purchased from Bioscan Industrie, Algeria.

### Methods

#### Preparation of the plant extract

After collecting the *Solanum nigrum* L. in June 2023 at the Province of Oued Souf, Algeria, the samples have been stored at the Herbarium of the Faculty of Biology at Ouargla University, with the assigned specimen number L.BIO30MN0001. Next; the samples were cleaned with tap water to remove any impurities, and repeatedly washed with distilled water. An extract of the *Solanum nigrum* plant has been prepared by adding 1000 mL of distilled water to 100 g of the sample and leaving it overnight. After that, it is filtered, stored in a bottle, and preserved at low temperatures.

#### Extraction of chitosan

**Figure 1:**
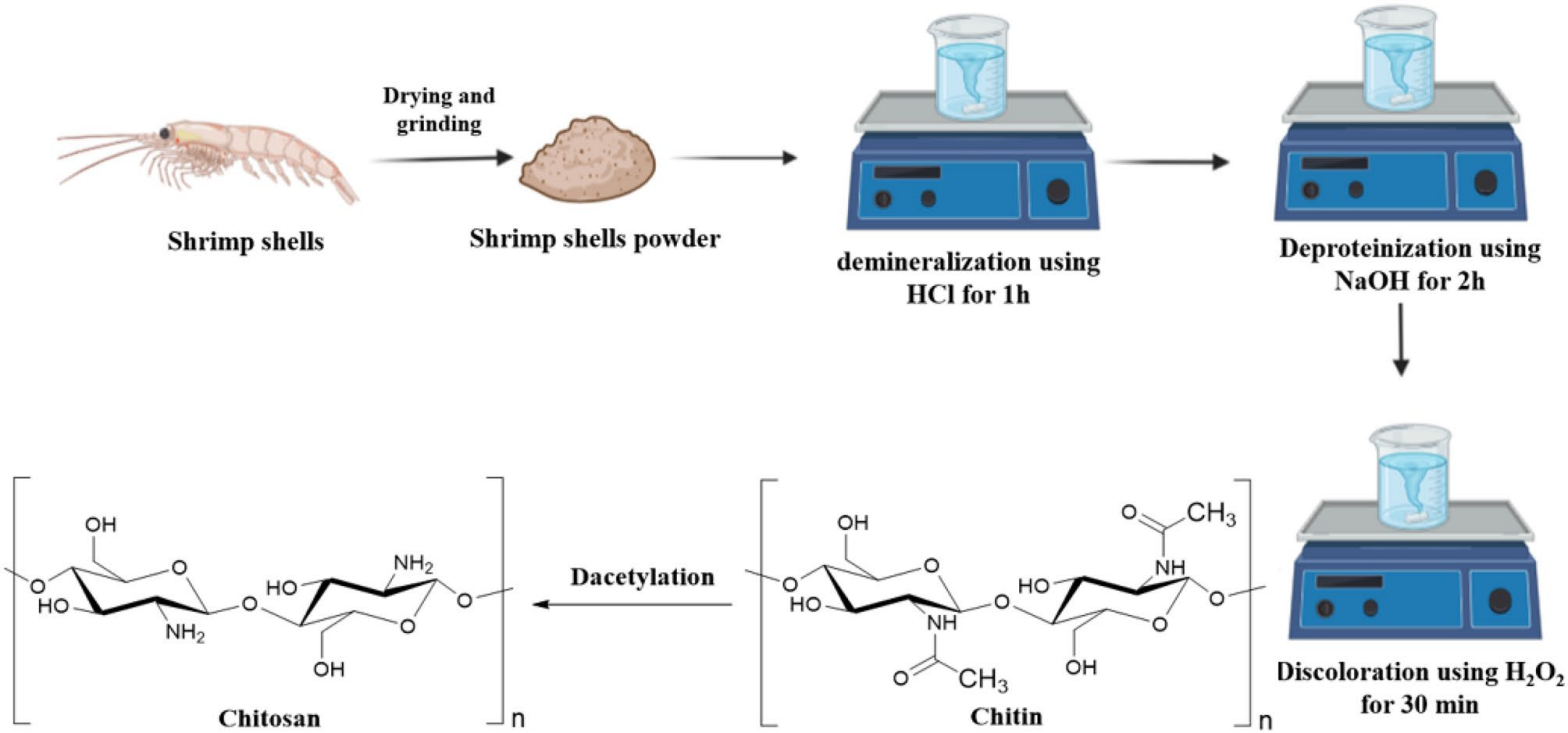
A Daiagram illustrating the procedures involved in the extraction of chitosan from shrimp shells.

Crushed shrimp shells were stored at − 20 °C for preservation. The demineralization process was carried out in accordance with the approach outlined by Kaya et al.^20^, where 30 g of shrimp shells were contacted with 450 mL of a 1 M HCl solution (a 1:15 solid-to-liquid ratio was used) at 40 °C for an hour. The resulting solid fraction was thoroughly rinsed with distilled water until it reached the pH of 7. Recovered chitin was then deproteinized by exposing it to a 1 M NaOH solution at 80 °C for 2 h. Chitin was then cleaned, filtered, and treated with a 10% H_2_O_2_ solution at 50 °C for 30 min to eliminate any remaining colorants. Then it was washed with distilled water until neutralization. Finally, decolored chitin was deacetylated by subjecting it to a 4-h treatment with a 50% NaOH solution at 100 °C. The resulting precipitate was cleaned with distilled water, and any chitosan that was still present was dried for 24 h at 50 °C in vacuum as shown in Fig. 1.

#### Biosynthesis of ZnO NPs

**Figure 2:**
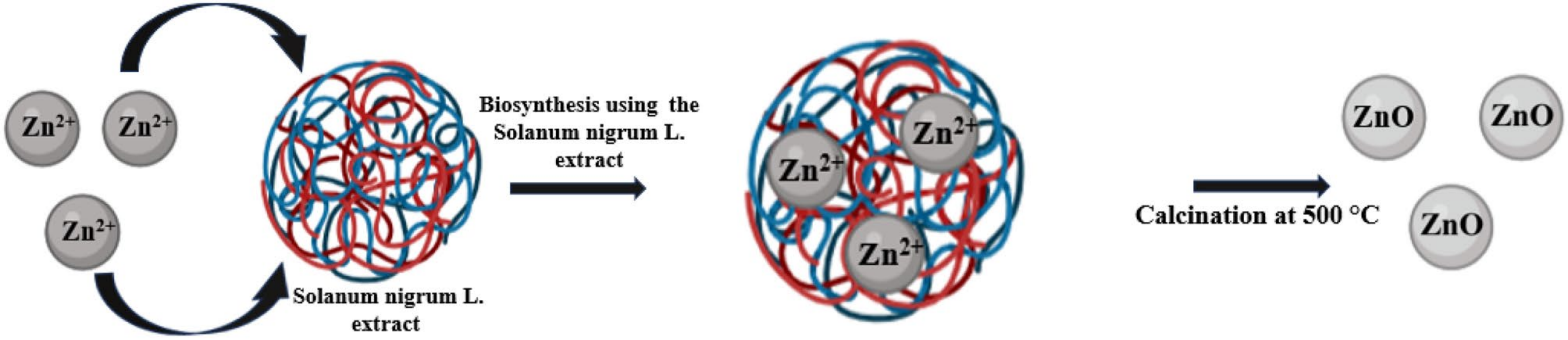
Schematic presentation showing the preparation of ZnO nanoparticles.

To synthesize ZnO NPs using the *Solanum nigrum* L. extract, about 0.1 M zinc chloride was mixed with 25 mL of the extract under vigorous stirring for 2 h. After the completion of the reaction, the formed dirty-colored precipitate was allowed to settle for 24 h. The precipitate was separated from the reaction solution by centrifugation at 6000 rpm for 15 min, repeatedly washed with deionized water to remove the impurities, and dried in an oven at 80 °C. To the powdered as-synthesized sample was then subjected to calcination in a muffle furnace at 400 °C for 4 h as presented in Fig. 2.

#### Preparation of chitosan–ZnO NPs

Zinc oxide (ZnO) powder (1 g) was dissolved in 1% acetic acid solution. (100 mL). 1 g of chitosan was then added to this solution. The resulting mixture was sonicated for 30 min and then magnetically stirred. Next, a gradual addition of a 1 M NaOH solution was continued until the pH of 9 was achieved. Afterwards, the mixture was heated in a water bath at a temperature between 40 and 80 °C for 3 h. The resulting precipitate was isolated by filtration and thoroughly rinsed using distilled water. The resulting material was dried for 1 h at 50 °C in an oven^21^.

Chitosan dissolves in acetic acid due to the protonation of its amino groups, rendering it soluble in water and yielding a viscous solution ideal for film formation. The ZnO nanoparticles undergo dispersion through ultrasound, effectively breaking up any agglomerates and ensuring a more homogeneous dispersion within the chitosan solution. Zinc oxide nanoparticles interact with chitosan via electrostatic interactions, hydrogen bonding, and van der Waals forces, crucial for stabilizing the nanoparticles within the chitosan matrix and preventing aggregation. Upon drying the precipitate, chitosan molecules draw closer, entrapping the zinc oxide nanoparticles within the polymer matrix. This drying phase culminates in the creation of a solid, seamless layer, wherein ZnO nanoparticles are evenly dispersed throughout the chitosan matrix.

#### Preparation of chitosan–ZnO/PVP nanocomposite

Preparation procedure of chitosan–ZnO/PVP nanocomposite (NC) was previously described^22^. To summarise, the following stages were included in the preparation procedure: At first, 110 mL of distilled water was mixed with 0.3 g of chitosan–ZnO and 0.3 g of Polyvinylpyrrolidone (PVP). For 20 min, the mixtures were left to stir at room temperature. Then, for 1 h at 45 °C, the chitosan–ZnO solution was ultrasonically dispersed. After that, the PVP solution was mixed with the chitosan–ZnO solution and left to stir for an hour. The next step was to ultrasonic disperse the mixture at 45 °C for 15 min after it was produced. Several centrifugation and washing cycles were performed on the solution to remove any leftover free PVP molecules. A day later, the modified chitosan–ZnO/PVP nanocomposites were baked at 60 °C to finish drying.

The chemical mechanism underlying the bonding of chitosan–ZnO within the PVP matrix primarily involves interactions at the molecular level. Initially, the chitosan–ZnO, due to their surface functional groups, engage in electrostatic interactions, hydrogen bonding, and van der Waals forces with the surrounding PVP polymer chains. These interactions facilitate the entrapment of chitosan–ZnO within the PVP matrix. The amine and hydroxyl groups present in chitosan form hydrogen bonds with carbonyl groups in PVP, leading to the establishment of intermolecular connections. Additionally, the polarity of the ZnO surface enables electrostatic attraction with the polar functional groups of PVP. Furthermore, van der Waals forces contribute to the overall stability of the composite by promoting interactions between non-polar regions of chitosan–ZnO and PVP. Through these cohesive forces, the chitosan–ZnO nanoparticles become effectively integrated within the PVP matrix, resulting in a synergistic composite material with enhanced properties.

#### Characterization

Chitosan, ZnO NPs, and the composite material of chitosan and ZnO NPs were analysed using different methods. It was obtained the absorption spectra of chitosan, ZnO NPs, chitosan–ZnO NPs, and Chitosan–ZnO/PVP using a UV–Vis spectrophotometer (Jasco, model V160 UV–Vis). The structural and chemical characteristics were investigated by scanning the FT-IR spectra in the 400–4000 cm^−1^ region using a Nicolet iS50 spectrometer. Using an acceleration voltage of 19 kV and a resolution of 200 Å, the samples were examined using a Carl Zeiss scanning electron microscope (FESEM, model Leo Supra 55) at magnifications ranging from 35 to 10,000. A Rigaku X-ray diffractometer (XRD), model Miniflex 600 XRD, was used to examine the crystalline structure of chitosan and ZnO NPs. Under a nitrogen environment and with a heating rate of 10 °C/min, the thermogravimetric analysis (TGA) was carried out using an SDT Q600 TG–DTA analyzer. Chitosan, ZnO NPs, the chitosan–ZnO NPs, and Chitosan–ZnO/PVP composite were thoroughly characterised using the aforementioned equipment, which yielded important insights into their structures and characteristics.

#### Antibacterial bioassay

Using the agar well diffusion method^23^, the antimicrobial efficacy of ZnO nanoparticles, chitosan–ZnO, and chitosan–ZnO/PVP nanocomposites was assessed against a set of four bacterial species. The microorganisms used in this study consist of two Gram-positive species, namely *Bacillus subtilis* (ATCC6633) and *Staphylococcus aureus* (ATCC6538), as well as two Gram-negative species, namely *Pseudomonas aeruginosa* (ATCC9027) and *Salmonella typhimurium* (ATCC14028). The culture plates were generated by using a sterile glass rod and 100 µL of a 24-h matured broth culture of the bacterial strains. Subsequently, a sterile cork borer was used to create six 6-mm wells in each Petri plate. Various concentrations of chitosan and chitosan–ZnO NPs were produced in acetic acid, specifically at concentrations of 0.2 mg/mL, 0.4 mg/mL, and 0.6 mg/mL. Three further doses of ZnO nanoparticles in DMSO were generated simultaneously (0.2 mg/mL, 0.4 mg/mL, and 0.6 mg/mL). The bactericidal activity of all these concentrations was assessed. Ciprofloxacin (CIP-5) (0.2 mg/mL, 0.4 mg/mL, and 0.6 mg/mL), a commonly used antibiotic, was used for the purpose of comparison. The plates were incubated at a temperature of 37 °C for a duration of 24 h to examine the zones of inhibition and facilitate bacterial growth.

#### Photocatalytic degradation

Under a 1000 W ultraviolet (UV) radiation lamp, the photocatalytic degradation of the toluidine blue (TB) dye in an aqueous solution was investigated using chitosan, ZnO NPs, and chitosan–ZnO NPs as photocatalysts. After preparing a 50 mL solution of the TB dye in distilled water with a concentration of 4 mM, 5 mg of the appropriate catalyst was added. At a constant temperature of 28 °C and a pH level of neutrality, the solution was maintained. The solution was then subjected to UV light at 5, 15, 30, 45, 60, 75, 90, 105, and 120 min intervals. The catalyst remained in a uniform suspension in the solution after ultracentrifugation. The photocatalytic process was monitored by measuring the absorbance at 631 nm using a spectrophotometer. The degradation of methylene blue (MB) dye was tested using the same experimental technique using chitosan, ZnO NPs, and chitosan–ZnO NPs as photocatalysts. The MB solution used had a concentration of 0.6 mM of the dye, and the absorbance measurements were recorded at 663 nm^24^.

##### Effect of pH on the photocatalytic degradation

Deionized water was used for the preparation of the pH solution with adjustment using 0.1 M HCl and NaOH solution.

##### Efficiency of dye photodegradation in subsequent cycles

Optimal conditions for the degradation of dyes utilising chitosan–ZnO and chitosan–ZnO/PVP NC were found to be 120 min and 0.005 grammes of catalyst, after an assessment of the influence of contact time and PH. Afterwards, chitosan–ZnO/PVP and reused chitosan–ZnO were tested for photocatalytic stability and efficiency. In order to do this, MB and TB dye solutions with a concentration of 0.6 µM were exposed to 5 mg of catalyst for 120 min while being irradiated with sunshine. The catalyst was extracted from the solution, rinsed, and dried at 100 °C after the dye concentration was evaluated by UV–Vis spectroscopy. The dried catalyst was then put back to use in further photocatalysis tests by adding it to a fresh dye solution (with a concentration of 0.6 µM). After the final experiment, the dried catalyst was subjected to XRD analysis in order to detect any structural changes.

#### Radical scavenger

##### 2,2ʹ‑Azino‑bis(3‑ethylbenzothiazoline‑6‑sulfonic acid) (ABTS)

The ABTS assay was performed following a method similar to Aouadi et al.^79^, with slight modifications. Different concentrations of chitosan, ZnO, chitosan–ZnO, chitosan–ZnO/PVP, and ascorbic acid were prepared for the assay. To prepare the test solution, 11.4 mg of ammonium persulfate and 64.3 mg of ABTS were dissolved in 25 mL of phosphate buffer (pH 7.4) containing 0.15 M NaCl, 0.1 M NaH2PO4, and 0.1 M Na2HPO4. This mixture was incubated for 30 min at 68 °C. The absorbance of the solution was measured at 734 nm and then adjusted by diluting with phosphate buffer until the absorbance reached 0.65. Subsequently, 0.04 mL of each prepared concentration (chitosan, ZnO, chitosan–ZnO, chitosan–ZnO/PVP, and ascorbic acid) was mixed with 1.96 mL of the diluted ABTS solution. The samples were then kept in the dark at 37 °C for 10 min before measuring their absorbance at 734 nm.

##### Diphenyl-1-picrylhydroxyl (DPPH) assay

For this study, was adapted Aouadi et al. method^79^ to conduct the DPPH assay. Was prepared various concentrations of chitosan, ZnO, chitosan–ZnO, chitosan–ZnO/PVP, and ascorbic acid. Each concentration was mixed with 2 mL of a 0.1 mM DPPH solution. The mixtures were then incubated at room temperature in the dark for 30 min to allow the reaction to occur. After incubation, the absorbance at 517 nm was measured using a double-beam UV–Visible spectrophotometer. The absorption value indicates the ability of the compounds to scavenge the DPPH radical, reflecting their antioxidant activity by showing a decrease in absorption at 517 nm. The radical scavenging activity was calculated as a percentage using the following formula^79^:

$$\text{Radical Scavenging Activity }\left(\text{\%}\right)=\frac{{A}_{control}-{A}_{sample}}{{A}_{control}},$$

where A_sample_ is the absorbance of the sample, and A_control_ is the absorbance of the control reaction. The IC_50_, or the concentration required for 50% inhibition of viability, was also determined.

#### In sillico toxicity studies

A web-based database called ProTox-II enables to predict the toxicological endpoints of the actual and hypothetical substances based on their chemical structure. To do this, the ProTox-II database employs the machine learning models that were trained on the data from the published literature (either in vitro or in vivo). The prediction of the input substance’s toxicity was done by using the trained machine learning models which depend on a database of structurally-related entities and similarity to known harmful chemicals. The approach used in this study integrated the knowledge about the chemical entity’s nature, the precise and comparative affinities, and the mechanism-based prediction in respect to various signalling and functioning processes.

- *Input variable* The user interface of ProTox-II is simple and self-explanatory. To assess a potential toxicity associated with a chemical entity, the user can draw the chemical structure of this entity or more simply type its chemical name. After requesting the website to show the predicted toxicity, this freely online available tool by default computes the request for the acute toxicity as well as the toxicity target^25^. Accordingly, names of our developed entities, i.e., chitosan, ZnO NPs, and chitosan–ZnO NPs, were written in order to anticipate any potential toxicities connected with a particular chemical composition.
- *Information output* On the results page, the average predicted LD_50_ in mg/kg wt was given in addition to the toxicity class (acute toxicity, organ toxicity, toxicity endpoints, toxicity pathways, and toxicity targets), the prediction accuracy, and the average similarity for three toxic compounds that are similar to each other and have known rodent oral toxicity values.

### Ethics approval

All authors approved.

## Results and discussion

### Characterization techniques

#### UV–Vis spectroscopy

**Figure 3:**
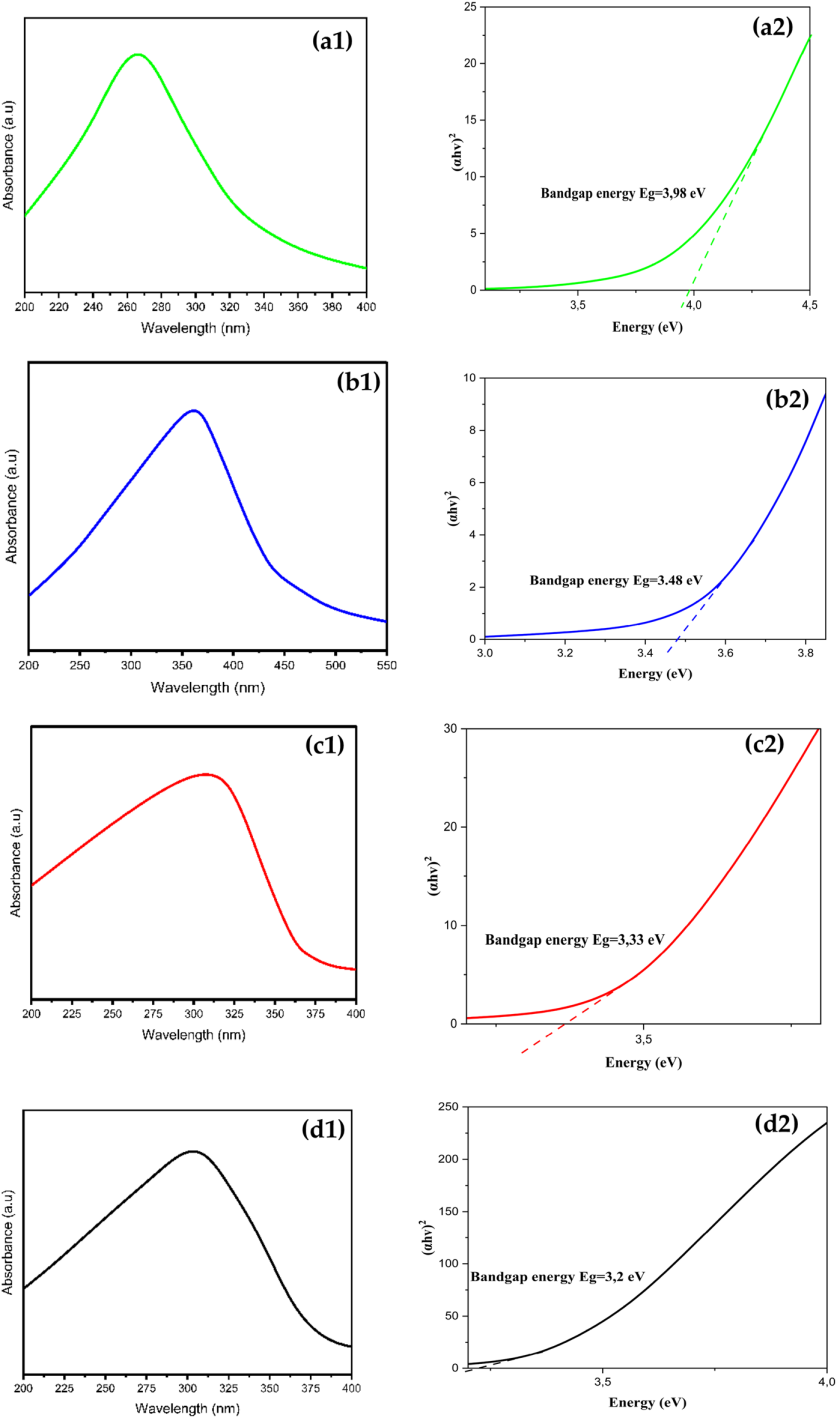
UV–Vis spectra and optical energy bandgap of (**a**) Chitosan, (**b**) ZnO NPs, (**c**) Chitosan–ZnO NPs and (**d**) Chitosan–ZnO/PVP.

Figure 3 displays the results of the analysis of the UV–Vis absorption spectra of several materials, including chitosan, ZnO NPs, chitosan–ZnO NPs, and chitosan–ZnO/PVP. In the case of chitosan, two initial absorption band is observed at 270 nm (Fig. 3a1). This band is attributed to the n–π* transition of the amino group present in chitosan. This means that the electrons in the amino group undergo an electronic transition from the non-bonding (n) orbital to the anti-bonding (π*) orbital upon light absorption at 270 nm^26^. Figure 3b1 represents the UV–Visible absorption spectrum of ZnO-NPs synthesized using of plant extract of *Solanum nigrum* L.^27^. This spectrum showed the absorption maximum at 356 nm which is characteristic to ZnO NPs. The shape of the UV–Visible spectrum is quite similar to the spectrum reported in a previous study^28^. As it could easily recognized from Fig. 3c1, the conjugation of ZnO with chitosan in chitosan–ZnO NPs causes a blue shift of the ZnO absorption maximum to a lower wavelength (310 nm compared with 356 nm). The reason for this is suggested to be due to the contact between ZnO and chitosan is lower (approximately 372 nm) compared to macrocrystalline ZnO^29^. Similarly, Fig. 3d1 shows another blue shift produced by Chitosan–ZnO/PVP where the absorption maximum was oberved at 312 nm. These last couple of observations are in line with the previous report by Kubo theory^30^. Kubo reported that as the nanoparticle’s diameter decreases, the absorption band will be blue-shifted. The peaks observed in the UV–Vis absorption spectra of chitosan, ZnO NPs and Chitosan–ZnO NPs are associated with specific electronic shifts of the functional groups or substances present in the samples^31^.

The band gap energy of Chitosan, ZnO NPs, chitosan–ZnO NPs, and chitosan–ZnO/PVP was calculated by plotting (hv)^2^ versus energy (eV) as shown in Fig. 3a2,b2,c2,d2. The band gap energies of Chitosan, ZnO NPs, chitosan–ZnO NPs and chitosan–ZnO/PVP are 3.98, 3.48, 3.33 and 3.20 eV, respectively. These results confirm that the use of chitosan and PVP to encapsulate ZnO NPs significantly affected the optical properties of ZnO NPs and reduced the energy band gap to a minimum.

#### FTIR spectroscopy

**Figure 4:**
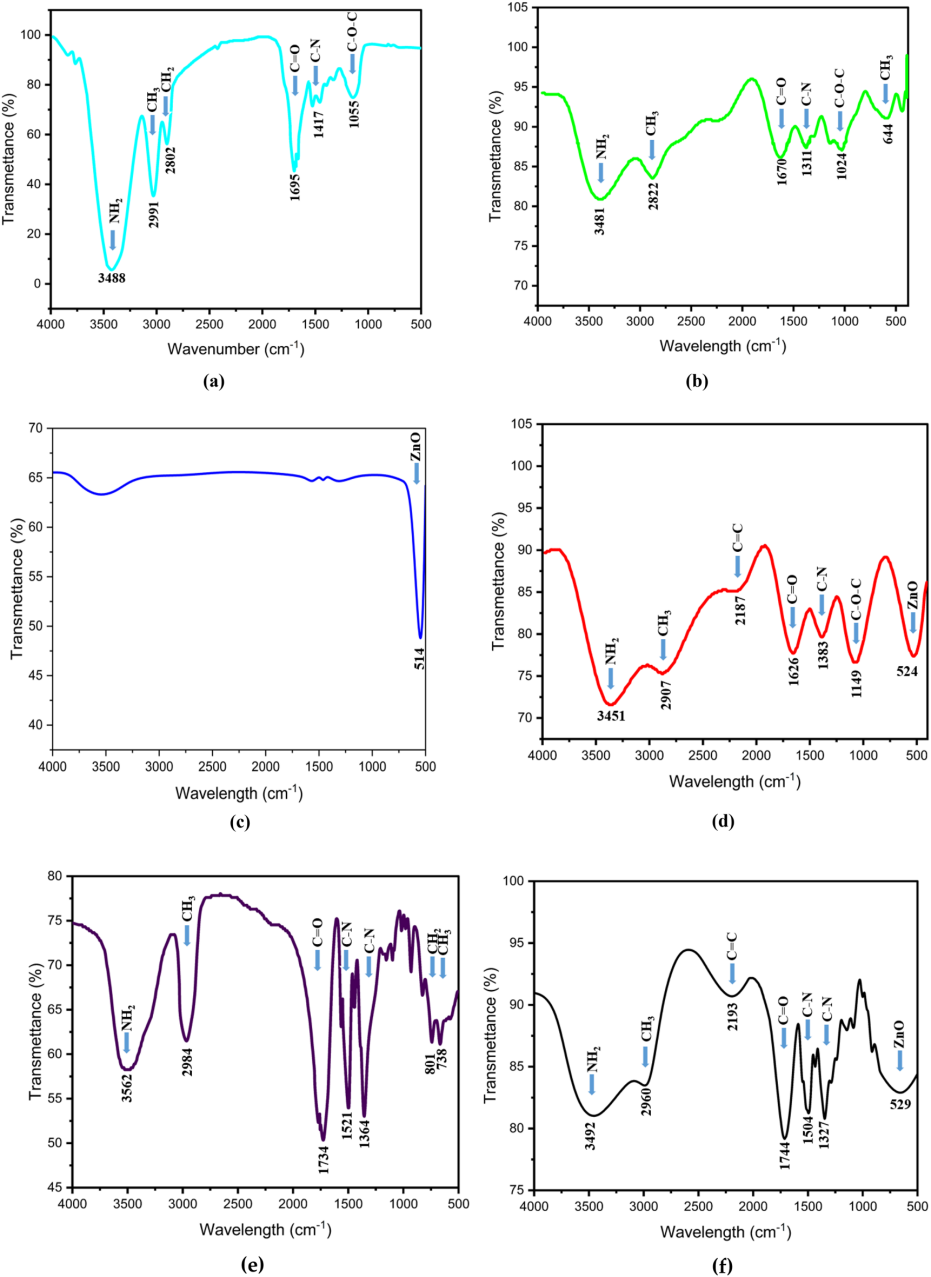
FTIR spectra of: (**a**) *S. nigrum* extract, (**b**) Chitosan, (**c**) PVP, (**d**) chitosan–ZnO, and (**e**) Chitosan–ZnO/PVP nanocomposite.

Figure 4a–f shows the FTIR spectra of *S. nigrum* extract, chitosan, ZnO NPs, PVP, chitosan–ZnO and chitosan–ZnO/PVP nanocomposite in the respective order. The peaks appear at the following absorption bands 3488, 2991, 2802, 1695, 1417, and 1055 cm^−1^ in Fig. 4a, which are due to the presence of the following functionalities, NH_2_, CH_3_, CH_2_, C=O, C–N and C–O–C^32–34^ respectively. Figure 4b shows that chitosan has a lot of functions in common with the plant extract with a new peak appearing at 644 cm^−1^ specific to CH_3_^35^. Figure 4c shows the FTIR spectra of ZnO NPs, where a characteristic absorption peak appears at 514 cm^−1^ for Zn=O. As shown in Fig. 4d–f, a new peak between 524 and 529 cm^−1^ appears in the spectra of chitosan–ZnO and chitosan–ZnO/PVP nanocomposite when compared with the infrared spectrum of chitosan. This peak corresponds to the symmetric stretching vibration of ZnO^36^. The characteristic peak of the NH group was shifted to a lower wavelength frequency of 3451 cm^−1^^37^. The presence of hydrogen bonds between ZnO and chitosan was confirmed by weakening the band at 1626 cm^−1^. The characteristic bands in Figs. 4d,f moved to lower wavenumbers, indicating conjugation between the hydroxyl, amino and amide groups of chitosan and zinc oxide^38^.

The FTIR spectrum of PVP (Fig. 4b) showed a band 1734 cm^−1^, which was attributed to the C=O intermediate stretching of the pyrrolidone ring^39–41^. The interaction between ZnO molecules and functional groups of chitosan and PVP was studied by Fourier transform infrared spectroscopy.

#### X-ray diffraction

**Figure 5:**
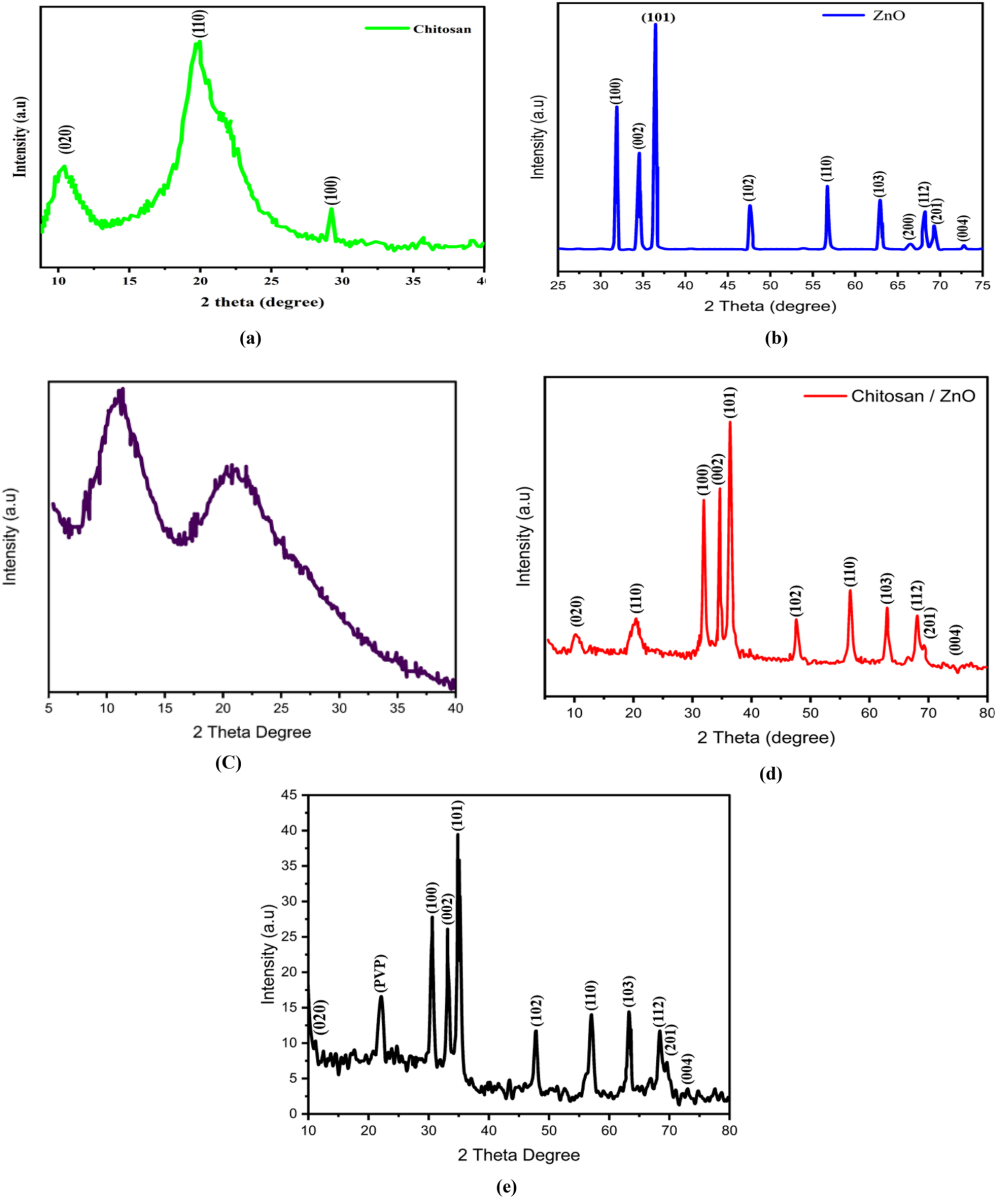
The X-ray diffraction (XRD) pattern of: (**a**) chitosan, (**b**) ZnO NPs, (**c**) PVP, (**d**) chitosan–ZnO NPs, and (**e**) Chitosan–ZnO/PVP.

Three diffraction peaks at 10.4°, 19.9°, and 29.1° are visible in the diffractogram of chitosan Fig. 5a–e. These peaks corresponded to the (020), (110), and (100) planes, respectively, of the crystal lattice. The peak at 10.4° had a lower intensity as compared to the intensity of the peak at 19.9°. This was likely due to the formation of the intramolecular hydrogen bonds during the deacetylation process, since these hydrogen bonds have an impact on the chitosan crystallinity and its structure^42^.

The crystallinity index was determined using the following formula:

$$Crystallinity\, index=\frac{({I}_{110}-{I}_{am})}{{I}_{110}}.$$

Here, I_*am*_ is a measure of the strength of the amorphous diffraction peak centered at 2θ = 10.4°, while I_110_ represents the maximum diffraction intensity at 2θ = 19.9°^43^. The degree of crystallinity in chitosan was indicated by the calculated crystallinity index value of 74.5%. Regarding the XRD pattern of ZnO NPs, several diffraction peaks were observed at the following angles: 32.1°, 34.9°, 36.8°, 47.2°, 56.7°, 62.3°, 66.5°, 67.7°, 68.8°, and 72.9°. These peaks corresponded to the (100), (002), (101), (102), (110), (103), (200), (112), (201), and (004) planes, respectively^44^, and indicated the presence of the well-crystallized ZnO structure of the hexagonal wurtzite crystal phase (JCPDS card no: 01-079-0205)^45^.

In the XRD pattern of chitosan–ZnO NPs, the characteristic peaks of chitosan at 10.4° and 19.9°were less prominent. The other diffraction peaks observed in the chitosan–ZnO NPs pattern corresponded to the (100), (002), (101), (102), (110), (103), (200), (112), (201), and (004) planes^46^. These peaks were sharper and stronger, indicating the high crystallinity of ZnO NPs. Based on the XRD analysis, it was concluded that the chitosan–ZnO complex was successfully formed at the nanoscale, as evidenced by the presence of the characteristic ZnO peaks and the change in the intensity of peaks in the difraction pattern of chitosan^47^. The XRD pattern showed distinct crystalline structures of PVP, ZnO, and chitosan in the chitosan–ZnO/PVP nanocomposite. This suggests that the chitosan–ZnO composites were entirely embedded in the PVP polymer matrix. So, the fact that the intensity peaks have been diminished indicates that the Chitosan–ZnO nanocomposite has been effectively combined with the polymeric chains. The results corroborate those of the UV–Visible and FTIR analyses.

The crystallite size was calculated using Scherrer’s equation

$$D=\frac{k\times\uplambda }{\beta \times \text{cos}(\theta )},$$

where k = constant = 0.91, λ is X-ray wavelength = 1.5418 Å, θ = Bragg’s angle, and β = The Full Width at Half Maximum (FWHM) corresponding to the highest intensity peak^44^.

The XRD diffraction data were utilized to deter-mine the crystallinity index and average crystallite size. As shown in Table 1, the crystallinity (%) values for the ZnO NPs, chitosan–ZnO and chitosan–ZnO/PVP were found to be 85.7%, 78.4% and 80.20%, respectively. The calculated crystallite size values were 15.3 nm, 17.8 nm and 30.1 nm for ZnO NPs, chitosan–ZnO and chitosan–ZnO/PVP, respectively.

**Table 1:** Summary for the different characteristics of the prepared ZnO NPs, chitosan–ZnO and chitosan–ZnO/PVP.

Sample	Crystallite size (nm)	Crystallinity (%)	Bandgap (eV)
ZnO NPs	15.3	85.70	3.48
Chitosan–ZnO	27.8	78.40	3.33
Chitosan–ZnO/PVP	30.1	80.20	3.20

#### SEM analysis

**Figure 6:**
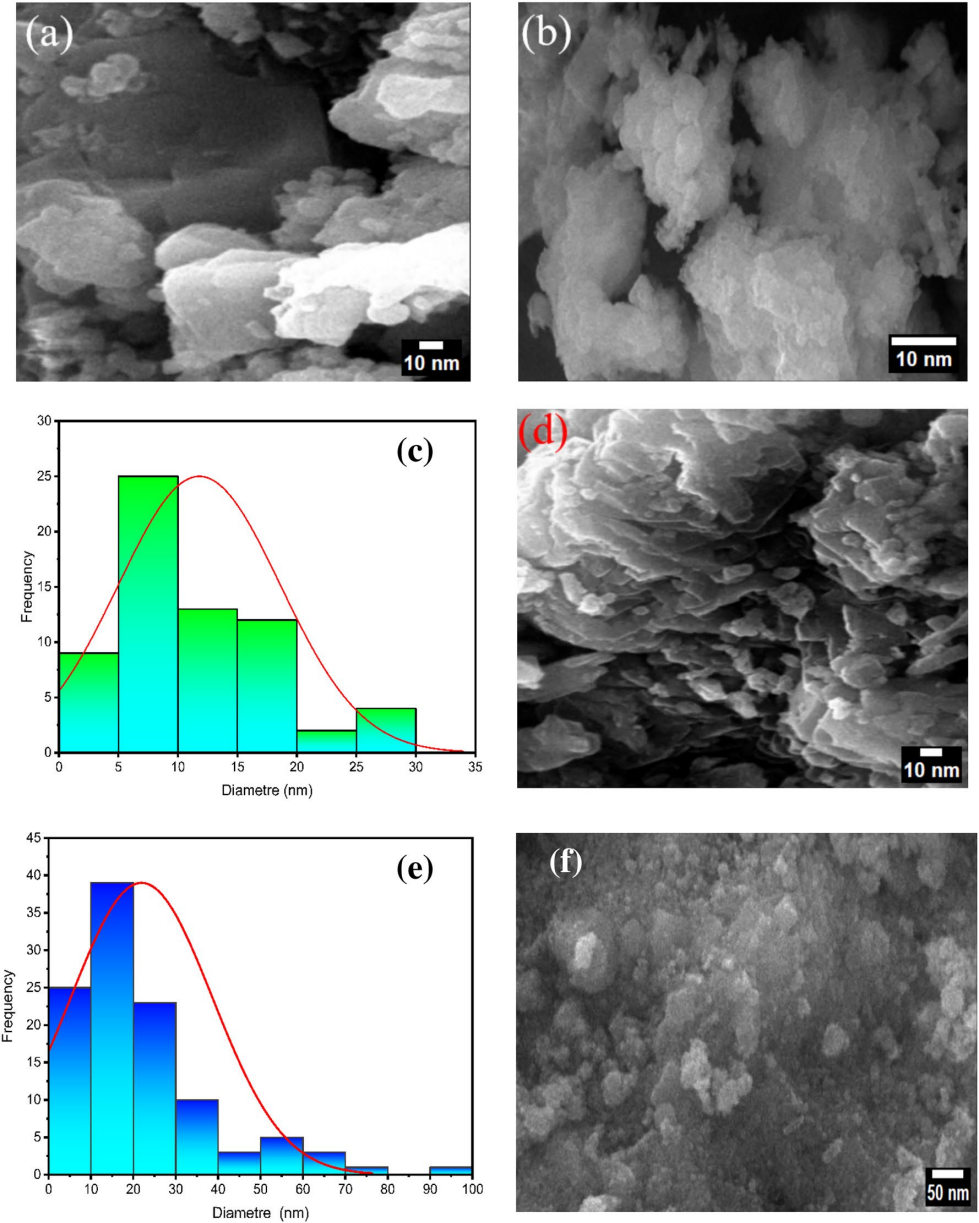
(**a**) The SEM image of chitosan, (**b,c**) the SEM image and the particle size distribution of ZnO NPs, (**d,e**) the SEM image and the particle size distribution of chitosan–ZnO NPs, and (**f**) the SEM image of chitosan–ZnO/PVP.

Chitosan, ZnO NPs, chitosan–ZnO NPs, and chitosan–ZnO/PVP were analysed using scanning electron microscopy (SEM) to look at their shape and size distribution. The results of these examinations are displayed in Fig. 6a for chitosan, Fig. 6b,c for ZnO NPs, Fig. 6d,e for chitosan–ZnO NPs and Fig. 6f for chitosan–ZnO/PVP.

It was established that chitosan derived from shrimp shells had a rough surface with many ridges. This shape was determined by various parameters, including the degree of deacetylation, the crystallinity index, the level of polymerization, and the temperature. ZnO NPs were spherical and elliptical agglomerates with few widely dispersed solitary NPs with an average diameter of 12 nm. Various parameters, such as the amount of the Zn(II) ions, the amount of chitosan, the temperature, and the pH, likely affected the resultant form of ZnO NPs. Finally, ZnO NPs coated with chitosan had variable shapes, as they were in the form of agglomerates of diverse forms, and this was possible due to the encapsulation of the ZnO NPs agglomerates with chitosan, which resulted in spherical, oval, and crystalline shapes. The encapsulation was successful upon comparing the morphology of ZnO NPs and chitosan–ZnO NPs. The average diameter of the latter NPs reached 21 nm.

According to the elemental analysis (Table 2), the atomic fractions of the elements in the different materials were as follows: In chitosan, carbon (C), oxygen (O), and nitrogen (N) were present at 44.19%, 34.38%, and 21.43%, respectively. In ZnO nanoparticles (NPs), zinc (Zn), oxygen (O), and carbon (C) were found at 73.42%, 24.76%, and 1.82%, respectively. For the chitosan–ZnO composite, the atomic fractions were 37.71% for carbon, 24.30% for oxygen, 15.16% for nitrogen, and 22.83% for zinc. Lastly, in the chitosan–ZnO/PVP composite, carbon, oxygen, nitrogen, and zinc were present at 40.57%, 26.12%, 16.78%, and 16.53%, respectively.

**Table 2:** The elemental composition of the chitosan, ZnO NPs, chitosan–ZnO and chitosan–ZnO/PVP.

Compound	Composition	
	Element	Atomic percentage (%)
Chitosan	C K	44.19
	O K	34.38
	N K	21.43
	Totals	100
ZnO NPs	Zn K	73.42
	O K	24.76
	C K	1.82
	Totals	100
Chitosan–ZnO NPs	C K	37.71
	O K	24.30
	N K	15.16
	Zn K	22.83
	Totals	100
Chitosan–ZnO/PVP	C K	40.57
	O K	26.12
	N K	16.78
	Zn K	16.53
	Totals	100

#### Thermal stability

**Figure 7:**
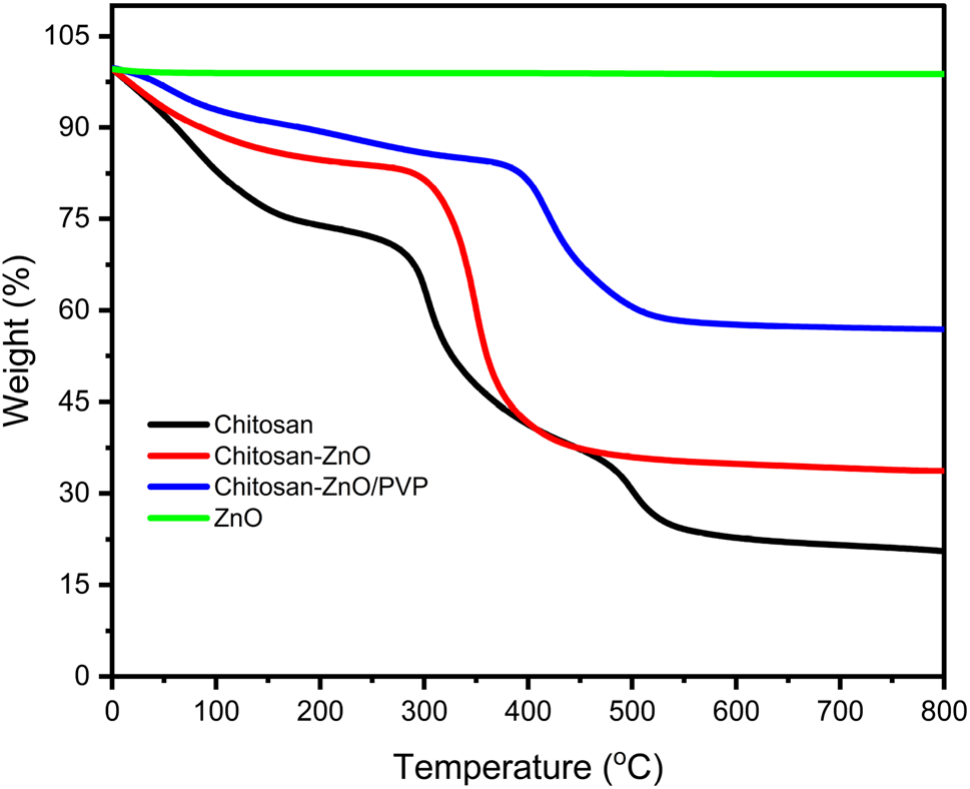
The TGA curves obtained for chitosan, ZnO NPs, chitosan–ZnO NPs, and chitosan–ZnO/PVP.

Figure 7 depicts the thermal stability curves of chitosan, ZnO NPs, chitosan–ZnO NPs, and chitosan–ZnO/PVP. The first weight loss occurred for all materials at temperatures less than 100 °C due to the moisture loss, followed by the second weight loss between 200 and 600 °C due to the loss of retained crystalline water in the case of ZnO NPs and chitosan–ZnO NPs. The initial disintegration of chitosan caused the weight loss at a temperature greater than 200 °C is due to the detachment of functional groups from the chitosan backbone. The process of decomposition of chitosan continued up to 800 °C, when its carbonization was completed and thermal stability was achieved. The thermal stabilities of ZnO NPs and chitosan–ZnO NPs were achieved at about 450 °C and 600 °C, respectively. Significant changes in the thermal properties of chitosan, ZnO NPs, and the composite material comprising chitosan and ZnO NPs were observed under conditions of elevated temperatures. The thermal breakdown temperature of chitosan was found to be lower than that of ZnO NPs and the composite material of chitosan–ZnO NPs. This observation suggests that chitosan exhibits a higher degree of heat sensitivity. The rapid breakdown of the polysaccharide constituents inside chitosan molecules in its structural framework leads to enhanced sensitivity. Polysaccharides, which are characterized by their elongated sugar chains, have a high susceptibility to heat degradation. The decomposition of chitosan molecules at elevated temperatures is attributed to the fracture or chemical interactions between *N*-acetylglucosamine and glucosamine units. In identical conditions, it was shown that both ZnO NPs and the chitosan–ZnO NP composite exhibited greater thermal stability. This suggests that the addition of chitosan did not significantly alter the thermal properties of the nanoparticles. The results show how different chitosan is when it comes to how it reacts to heat and what that could mean for materials that are sensitive to heat.

It was obvious that the thermal constant of ZnO NPs produced with the aid of chitosan was quite high, which led to the enhancement of chitosan thermal stability when ZnO NPs were coated with it. Another research also observed similar results in the case of chitosan–ZnO^48^.

The thermal characteristics of the nanocomposites were investigated through Thermogravimetric Analysis (TGA), and the thermo-gravimetric responses of the chitosan–ZnO/PVP nanocomposites are depicted in Fig. 7. The initial weight loss is observed within the temperature range of room temperature to 100 °C, attributed to the dehydration of water molecules present in all Chitosan and PVP-based materials^49^.

The thermal behavior of the chitosan–ZnO/PVP nanocomposites revealed two distinct stages of weight loss occurring between 100–410 and 410–590 °C. These stages are associated with the carbonization of PVP and polysaccharides. Notably, the weight derivative of the Chitosan–ZnO/PVP nanocomposite indicates a 41% weight loss. This suggests a significant enhancement in the thermal stability of the chitosan–ZnO nanocomposite^50^.

### Photocatalytic degradation

#### Photocatalytic degradation of MB and TB dye

The best approach to the elimination of the synthetic dyes from wastewaters is their adsorption. For several metal ions and organic pigments, a high amino functionality content of chitosan offers an attractive adsorption characteristics. Chitosan functional groups can interact with MB and TB molecules through the covalent, electrostatic, and hydrogen bonding interactions. The greater acetylation of chitosan, its grafting (the introduction of functional groups), or crosslinking with other polymers may improve the adsorption properties toward various contaminants in wastewaters, and increase its resistance to harsh media conditions. The adsorption capacity of chitosan is large when the DD value is high, hence the DD of chitosan is important.

**Figure 8:**
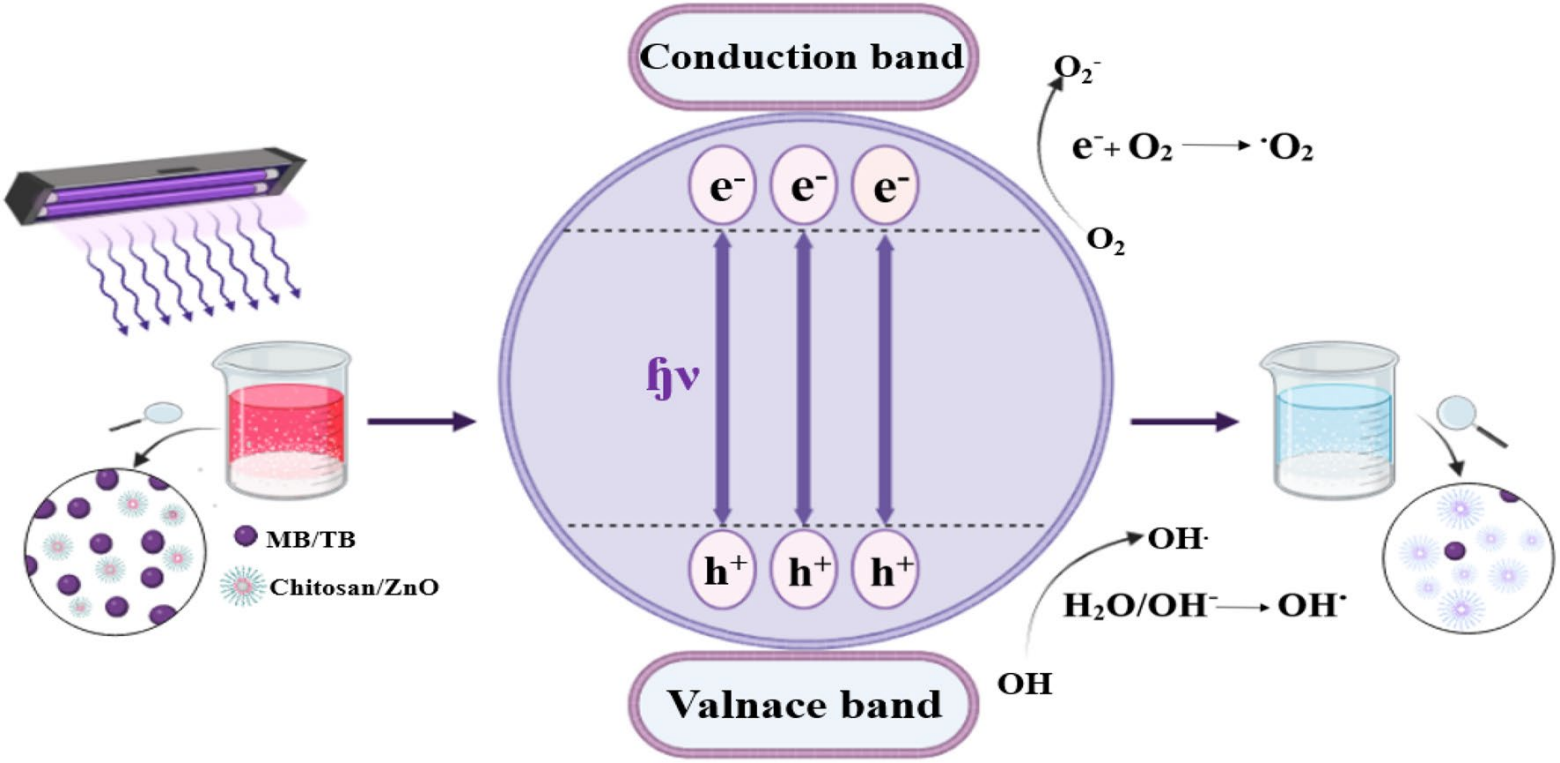
The diagram illustrating the mechanism by which ZnO NPsis utilized to photodegrade the MB and TB dye.

In the present work, the effectiveness of ZnO NPs, and chitosan–ZnO NPs, and Chitosan–ZnO/PVP in removing MB was 97.4%, 99.6%, and 100%, respectively, within 120 min as seen in Table 2 and Fig. 8. In the case of TB, the removal efficacy of this dye for ZnO NPs, chitosan–ZnO NPs, and Chitosan–ZnO/PVP was 96.8%, 99.5%, and 100%, respectively, also within 120 min as showed in Table 3.

**Table 3:** The comparison of the MB and TB dyes removal efficiency results using chitosan, ZnO NPs, chitosan–ZnO NPs, and chitosan–ZnO/PVP.

Material	Azo dye	Time (min)	Dye removal (%)	Reference
Chitosan	Methylene blue	60	93.2	^57^
Chitosan	Methylene blue	60	84.9	^58^
Chitosan	Methyl red	60	90.9	^59^
ZnO NPs	Methylene blue	120	78	^60^
ZnO NPs	Methylene blue	90	87.5	^61^
ZnO NPs	Methylene blue	120	97	^62^
ZnO NPs	Methylene blue	120	97.4	This study
	Toluidine blue		96.8	
Chitosan–ZnO NPs	Methylene Blue	120	99.6	This study
	Toluidine blue		99.46	
Chitosan–ZnO/PVP	Methylene blue	120	100	This study
	Toluidine blue		100	

Table 3 compares the efficiency of chitosan and ZnO NPs in removing the AZO dyes from diverse sources. When exposed to light, ZnO NPs can break down some dangerous chemical molecules^51^. The properties of ZnO NPs and the arrangement of the active species produced in the reaction media dictate the beneficial photocatalytic process. To examine the potential function of various active species in the photodegradation of Azo dyes, ZnO NPs were applied in radical scavenging tests. When the Azo dye is exposed to light, its breakdown is discovered to involve several substances, including isopropanol, formic acid, oxalic acid, and ascorbic acid^52^. However, in the present work, no extreme scavengers were used, just model organic pollutants, i.e., the MB and TB dyes, were utilized to assess the photocatalytic activity of ZnO NPs when exposed to UV radiation. Both the MB and TB dyes are toxic, carcinogenic, and non-biodegradable, posing serious issues for the human health and the environment^53^. Therefore, the development of the effective and ecologically friendly procedure for their degradation and removal in wastewaters is of special importance. In particular, the photocatalytic degradation is recommended because it benefits from the complete mineralization of the pigments into direct, non-toxic species and much lower processing costs. Comparing the concentration of MB or TB dye in the aqueous solution after the photolysis test allowed researchers to determine how much adsorption had^54^.

The MB and TB dyes were catalytically reduced when the UV light was used. The photocatalytic degradation efficiency (%) of both dyes was calculated using the equation^55^.

$$Degradation\, ratio (\%) =\frac{\left({C}_{0}-{C}_{t}\right)}{{C}_{0}}\times 100,$$

where *C*_*t*_ is the current concentration, and *C*_0_ is the starting concentration of MB or TB.

In the process of photocatalysis, the electrical structure of zinc oxide nanoparticles (ZnO NPs) is the most important thing. Zinc oxide (ZnO) is a semiconductor because it has two energy bands that can be easily identified: the valence band (VB) and the conduction band (CB). In the valence band (VB), electrons are usually in lower energy states and are attached to zinc (Zn) and oxygen (O) atoms. When ZnO is exposed to light, photons with energy equal to or greater than the band gap can be absorbed. This makes it easier for electrons to move from the valence band (VB) to the conduction band (CB). The process creates “conduction band electrons” and makes “valence band holes” inside the valence band at the same time. During photocatalysis, the presence of charged particles, such as CB electrons (*e*^−^) and VB holes (*h*^+^), is a key part of how reactive species (VB), *h*^+^, O_2_, and OH are made^41^. On the surface of the ZnO nanoparticles, these charged particles help make a number of oxidation and reduction processes happen (Eqs. 1–8). As a result of the energy being higher than the band gap of ZnO, the CB electrons (e) and the VB holes (h+) are encouraged to be developed. The adsorbed MB and TB dyes may be directly oxidized by the photogenerated holes on the VB or it may react directly with the hydroxyl (OH). In the CB, the photoelectrons can change O_2_ adsorbed on the surface of ZnO NPs into the superoxide radicals (O_2_). For this reason, MB and TB dyes can be degraded by the photocatalysis with the production of both OH and O_2_^56^ as illustrated in (Fig. 8).

1 $${\text{ZnO}}{-}^{hv}\to {\text{ZnO}}\left({e}^{-}\left(CB\right)\right)+{\text{ZnO}}\left({h}^{+}\left(VB\right)\right),$$

2 $${\text{ZnO}}\left({h}^{+}\left(VB\right)\right)+ {\text{H}}_{2}{\text{O}}\to {\text{ZnO}}+ {\text{H}}^{+}+ {\text{OH}}^{+},$$

3 $${\text{ZnO}}\left({e}^{-}\left(CB\right)\right)+ {\text{O}}_{2}\to {\text{ZnO}}+ {\text{O}}_{2}^{-.},$$

4 $${\text{H}}^{+}+ {\text{O}}_{2}^{-.}\to {\text{OH}}_{2}^{.},$$

5 $$2{\text{OH}}_{2}^{.}\to {\text{H}}_{2}{\text{O}}_{2}+{\text{O}}_{2},$$

6 $${\text{H}}_{2}{\text{O}}_{2}{-}^{hv}\to {\text{OH}}^{.},$$

7 $$Methylene\, blue +{\text{OH}}^{.} \to Degradation\, products+{\text{CO}}_{2}+{\text{H}}_{2},$$

8 $$Toluidine\, Blue+{\text{OH}}^{.} \to Degradation\, products+{\text{CO}}_{2}+ {\text{H}}_{2}{\text{O}}.$$

#### Recycling performance

A photocatalyst’s efficacy in water remediation applications is dependent on its separability and reusability^63^.

**Figure 9:**
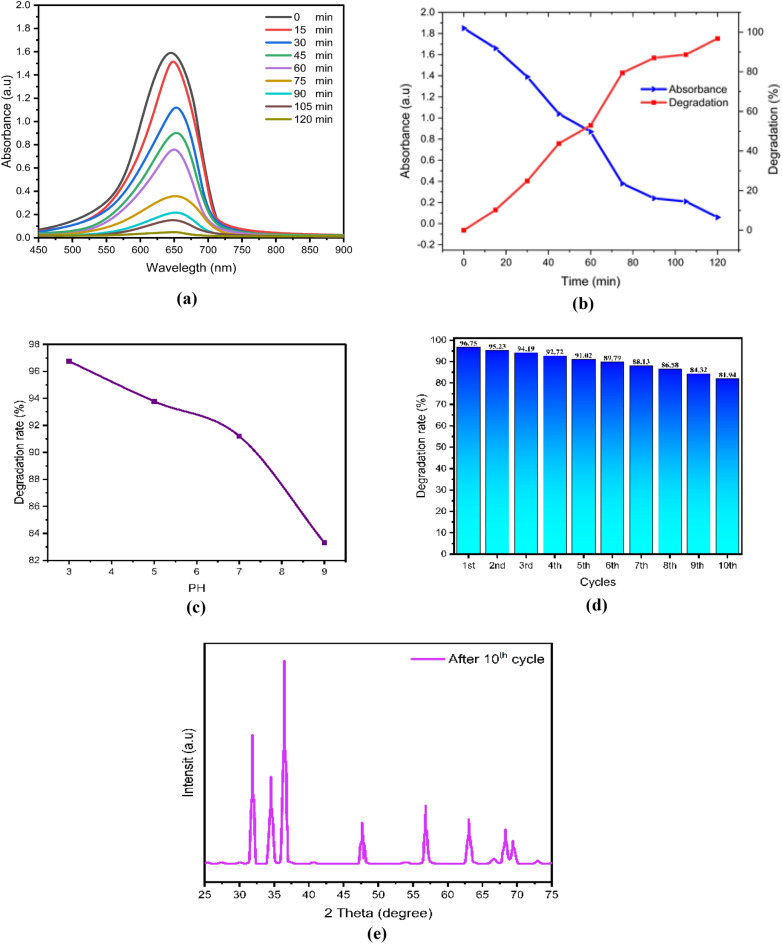

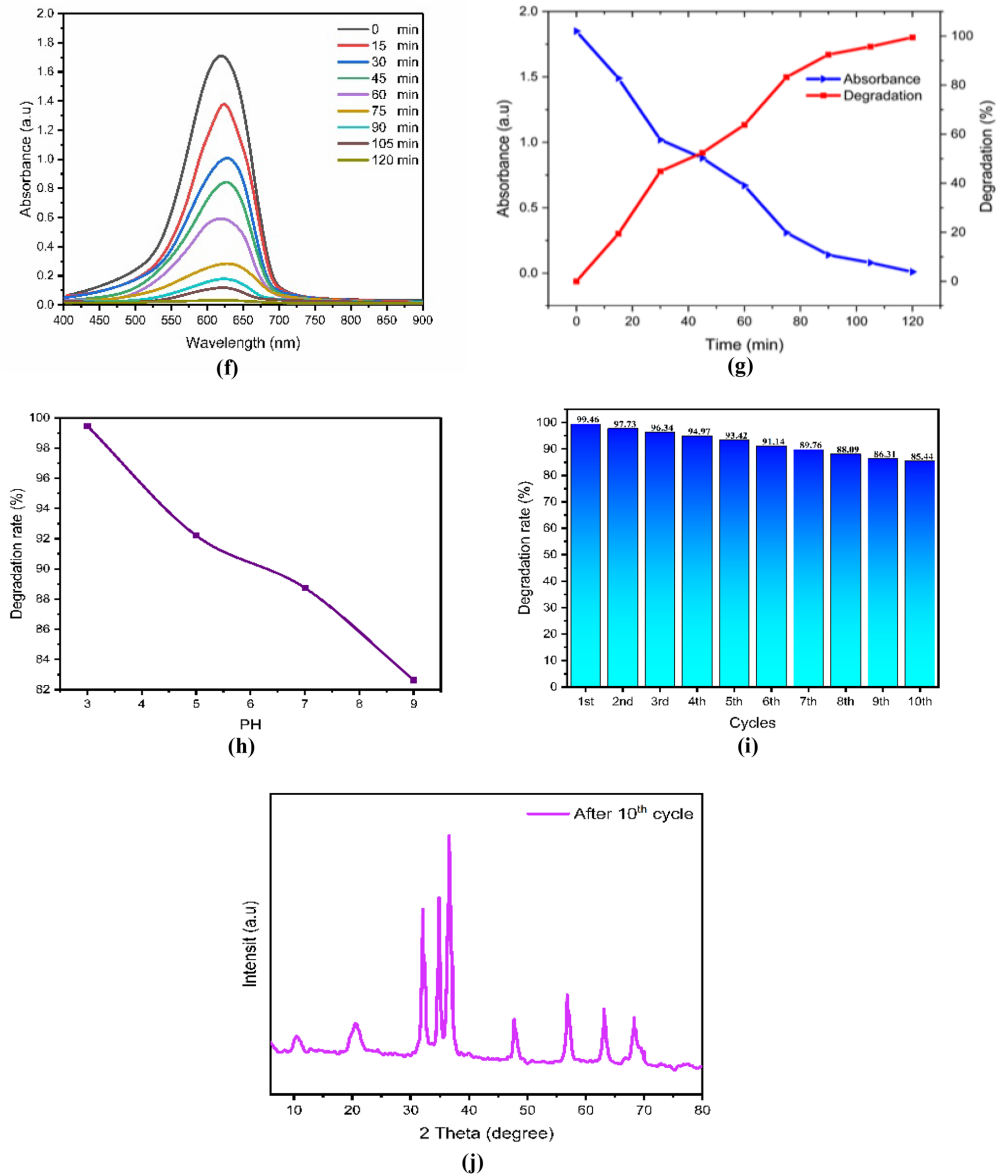

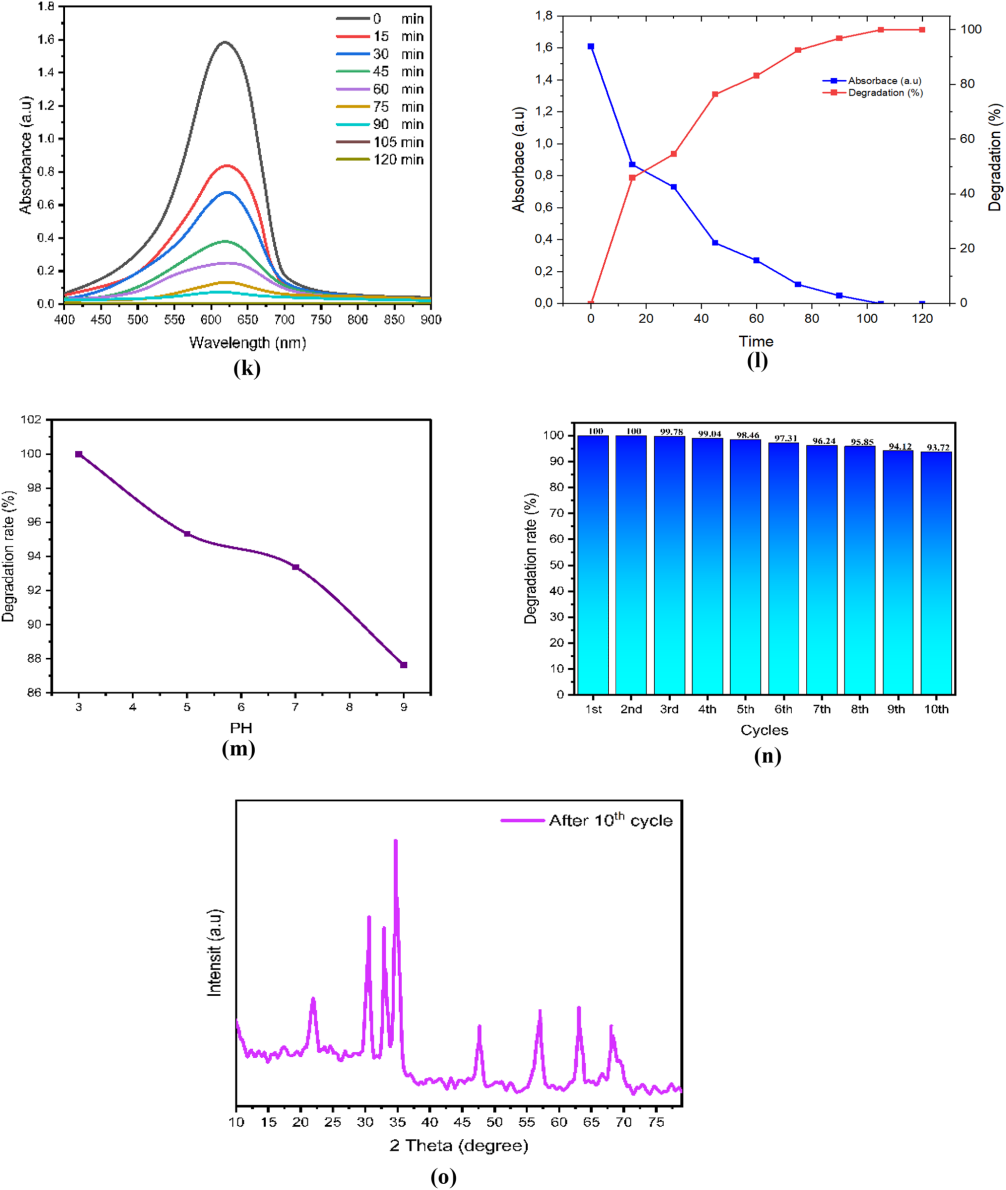
The photodegradation efficiency, the reaction time, PH, cycles, DXR after 10 cycles and the Toluidine Blue (TB) dye degradation percentage: (**a–e**) ZnO NPs; (**f–j**) chitosan–ZnO NPs; and (**k–o**) chitosan–ZnO/PVP.

**Figure 10:**
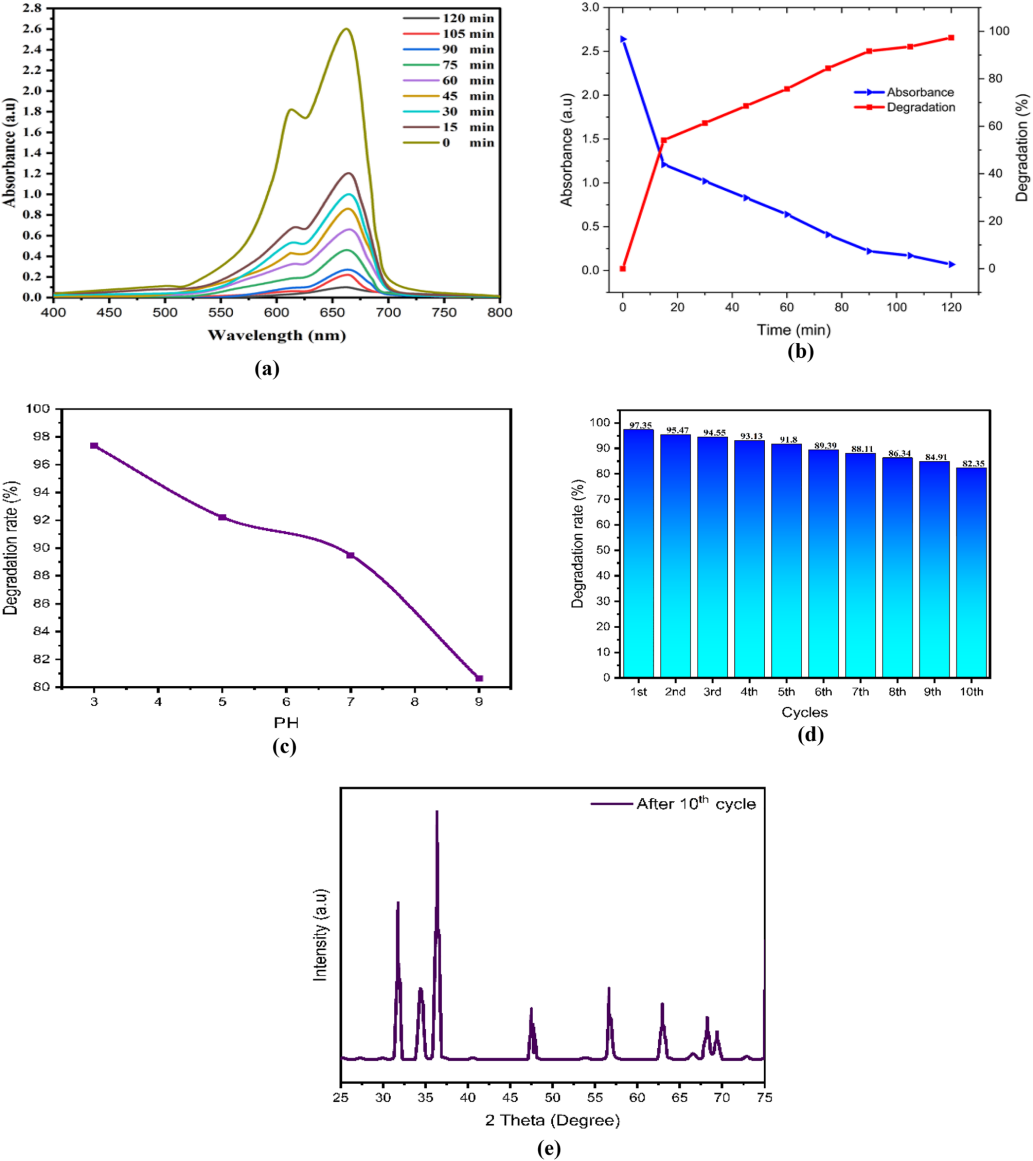

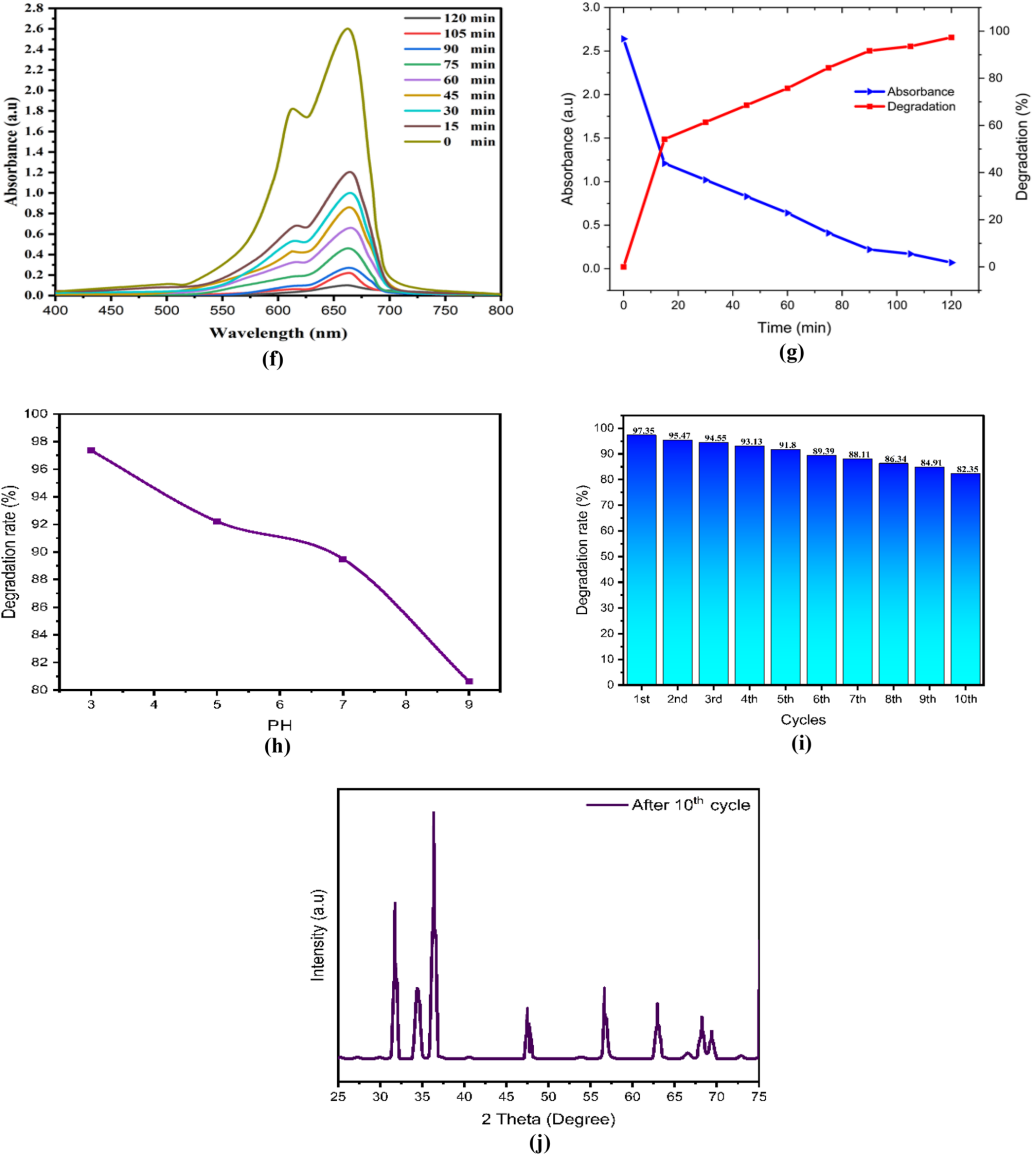

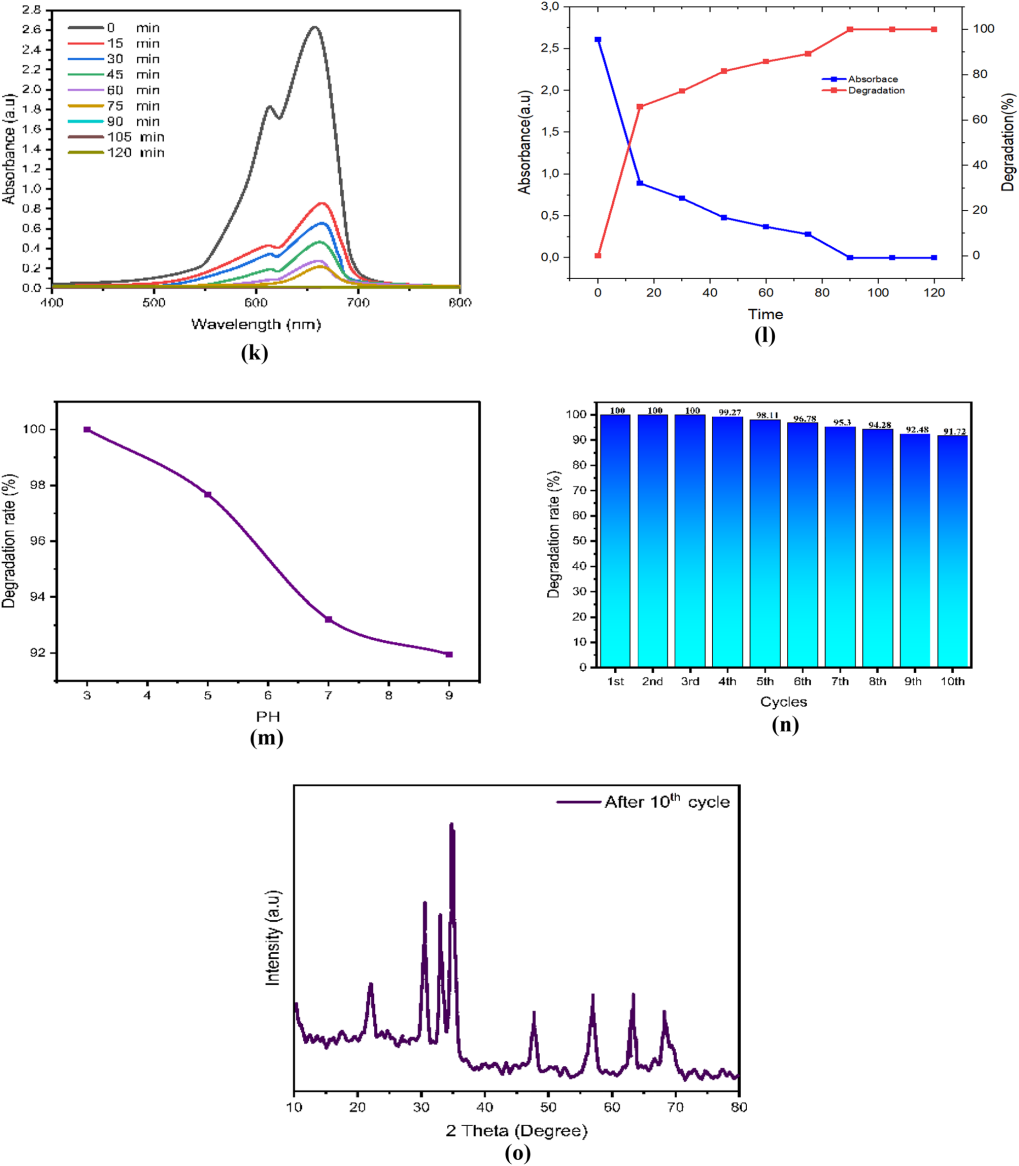
The photodegradation efficiency, the reaction time, PH, cycles, DXR after 10th cycles and the Methylene Blue (MB) dye degradation percentage. (**a–e**) ZnO NPs; (**f–j**) chitosan–ZnO NPs; and (**k–o**) chitosan–ZnO/PVP.

After drying, the ZnO, chitosan–ZnO, and chitosan–ZnO/PVP photocatalysts were re-used in a second photocatalysis cycle with identical parameters to determine their recyclability. Figure 10e,j,o for MB dye and Fig. 9e,j,o for TB dye show the results for ZnO, chitosan–ZnO, and chitosan–ZnO/PVP in terms of the photocatalysts’ recyclability across 10 consecutive cycles. In terms of degrading MB and TB dyes, the results show that the generated ZnO, chitosan–ZnO, and chitosan–ZnO/PVP photocatalysts are very effective and may be reused. Still, after 10 cycles, the photocatalytic activity started to drop somewhat. Reasons for this might include catalyst depletion during centrifugation and washing, or the adsorption of intermediate species formed during photocatalysis^64^.

After 10 photocatalytic cycles, the XRD results showed that the ZnO, Chitosan–ZnO, and Chitosan–ZnO/PVP photocatalyst maintained its critical XRD diffraction peaks, as shown in the figures. So, the ZnO, chitosan–ZnO, and Chitosan–ZnO/PVP photocatalysts’ diffraction peaks were unaltered by the catalytic material.

#### Effect of pH

One of the main factors that influence the rate of degradation of some organic compound pollutants is the pH value since it dictates the surface charge properties of the catalyst and size of aggregates it forms^65^.

In this study, Figs. 9a–o and 10a–o represent a photodegradation efficiency, the reaction time, PH, cycles, DXR after 10 cycles the MB and the TB dye degradation percentage, where Figs. 9 and 10a–e ZnO NPs; Figs. 9 and 10f–j chitosan–ZnO NPs; and Figs. 9 and 10k–o chitosan–ZnO/PVP.

The effect of pH, on the degradation of MB and TB dyes was conducted with pH values of 3, 5, 7, and 9. The highest performance of the degradation of MB and TB dyes was achieved at pH 3, followed by pH 5, pH 7, and pH 9, as shown in Figs. 9c,h,m and 10c,h,m for MB and TB dyes respectively. As the pH value increases, the percentage of MB and TB degradation will be decreased. Semiconductor metal oxide usually exhibits an amphoteric behavior, which will influence the surface-charge (pzc) properties of the catalyst when the reactions occur on the surface of the semiconductor^66^.

Thus, since MB and TB are negatively charged anions, they tend to accumulate on the surfaces of ZnO, chitosan–ZnO, and chitosan–ZnO/PVP catalysts when the pH of the solution is low. This is because these catalysts are naturally positively charged at pH values lower than pHpzc. This happens because the electrostatic interaction between the MB and TB dye molecules and the ZnO, chitosan–ZnO, and chitosan–ZnO/PVP catalysts increases, leading to faster dye degradation at low pH values.

The results showed that chitosan–ZnO/PVP had a greater effectiveness in removing both the MB and TB dyes from water than the effectiveness of chitosan–ZnO or ZnO NPs.

### Antibacterial bioassay

**Figure 11:**
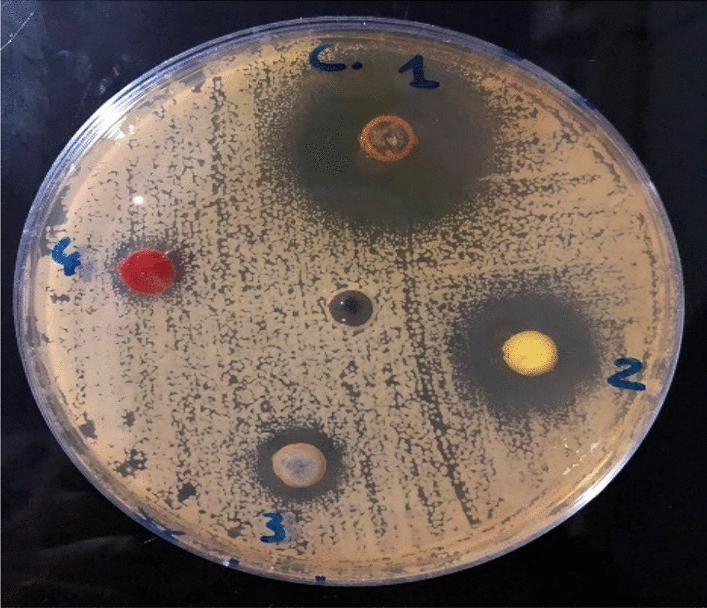
The antibacterial activity effect of the prepared samples.

Table 4 lists the results of the antibacterial action of chitosan, ZnO NPs, chitosan–ZnO NPs, and Chitosan–ZnO/PVP against the Gramme (+) bacteria *Staphylococcus aureus and Staphulococcus aureus* and the Gramme (−) bacteria *Pseudomonas aeruginosa* and *Salmonella typhimurium* (Fig. 11). It was established that when the chitosan concentration increased, the inhibitory zone of the compound widened. The antibacterial action of chitosan is mostly influenced by the type of bacteria, their stage of development, their concentration, their molecular weight, the pH and temperature of the solution, and their molecular weight^67^. It is recognized that the negatively charged molecules on the bacterial cell membranes interact with the positively charged protonated amino groups of chitosan, killing the bacterial cells^68^. Silva et al*.*^69^ observed a complete antibacterial barrier after applying Buriti oil to chitosan films, while Devliger et al.^70^ calculated the antibacterial efficacy of commercial chitosan against a number of putrefactive microorganisms with a high percentage of deacetylation (94%) and a lower wt. The researchers noted that while Gram (+) bacteria diffferently responded to chitosan, the Gram (−) bacteria were particularly susceptible to it.

**Table 4:** The zone of inhibition of chitosan, ZnO NPs, chitosan–ZnO NPs, and Chitosan–ZnO/PVP at different doses against the studied bacteria strains.

Sample	Zone of inhibition (mm)				
	Conc (mg/ml)	Gram-positive		Gram-negative	
		*Bacillus subtilis*	*Staphulococcus Aureus*	*Pseudomonas aeruginosa*	*Salmonella Typhimuruim*
ZnO NPs	0.2	14 ± 0.25	17 ± 0.33	13 ± 0.13	12 ± 0.25
	0.4	16 ± 0.21	18 ± 0.25	14 ± 0.54	13 ± 0.25
	0.6	17 ± 0.33	19 ± 0.25	15 ± 0.21	15 ± 0.15
Chitosan–ZnO NPs	0.2	14 ± 0.25	21 ± 0.15	14 ± 0.75	15 ± 0.33
	0.4	21 ± 0.30	23 ± 0.66	16 ± 0.34	17 ± 0.66
	0.6	21 ± 0.30	24 ± 0.80	18 ± 0.63	19 ± 0.21
Chitosan–ZnO/PVP	0.2	20 ± 0.39	22 ± 0.34	16 ± 0.23	18 ± 0.67
	0.4	21 ± 0.21	23 ± 0.25	18 ± 0.44	19 ± 0.33
	0.6	22 ± 0.34	25 ± 0.21	19 ± 0.21	22 ± 0.39
Ciprofloxacin (CIP-5)	0.2	21 ± 0.15	21 ± 0.30	18 ± 0.25	18 ± 0.33
	0.4	22 ± 0.21	23 ± 0.13	20 ± 0.25	21 ± 0.17
	0.6	22 ± 0.73	25 ± 0.33	22 ± 0.67	24 ± 0.14

ZnO NPs was found to havee a significant antibacterial effect against the Gram (+) bacteria but against the Gram (−) bacteria it was a lower efficacy (Table 4). The kind of the cell wall, the binding sites for ZnO NPs on the cell surface, and how ZnO NPs interact with the internal cell components decide about the antimicrobial effect of this nanomaterial. The Gramme (+) bacteria responded to the ZnO NPs antibacterial properties more readily than the Gramme (−) bacteria. This likely resulted from the contrast between Gramme (+) and Gramme (−) microorganisms in the outer membrane^71^.

Based on the given results, there is no doubt that chitosan exhibited the antibacterial properties, but it was less potent than ZnO NPs. Chitosan–ZnO NPs and chitosan–ZnO/PVP were characterised by a much higher antibacterial activity than chitosan alone, with the largest increase in the antibacterial activity against the Gram-positive or Gram-negative bacteria. The increase in the biological activity of both was likely associated with both the ZnO–chitosan-, and PVP-induced inhibition of the bacterial growth. However, compared with either chitosan alone, the presence of a small amount of ZnO with chitosan was sufficient to increase the antibacterial activity. Several literary reviews^72^ reported that ZnO NPs exhibit an antibacterial effect, but the specific mechanism is still under discussion. It was demonstrated that the release of the Zn(II) ions during the dissolution process is the primary mechanism responsible for the antibacterial activity of ZnO NPs^73^. The microorganisms are also damaged and killed or destroyed by active free radicals that are formed on the surface of ZnO.

**Figure 12:**
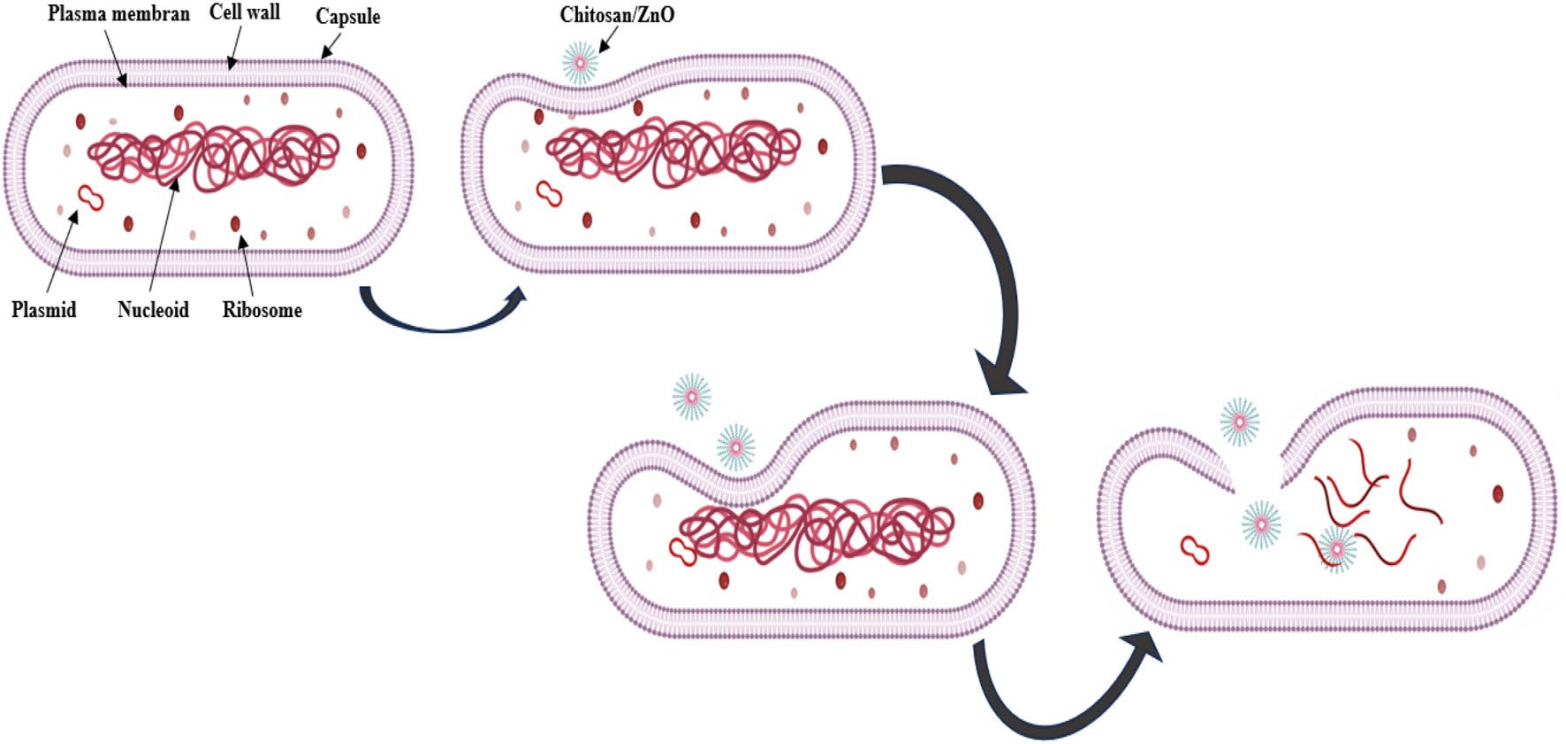
The probable mechanism of the antibacterial activity.

The Gram-positive bacterial strain *Staphylococcus aureus* was the most susceptible to the antibacterial action of the studied nanoparticles. Several critical factors likely affected this effect and its magnitude. The changes in the chemical composition and the structure of the cell membranes, and particularly the characteristics of the cell walls were possibly responsible for the variations in the antibacterial activity of the NPs against the two different types of bacteria studied here. The periplasmic region, which acts as a barrier between the outer and inner membranes and allows the entry of the inhibitory compounds, is easily solubilized by the thin outer membrane (Fig. 12)^74^.

The present study observed that the antibacterial efficacy of chitosan and zinc oxide nanoparticles exhibited similar results to those reported in earlier research. Additionally, it was seen that the antibacterial activity of chitosan-coated zinc oxide nanoparticles surpassed the outcomes reached in prior investigations, as shown in Table 5. The aforementioned conclusions were drawn from the data that was gathered. This finding illustrates the successful integration of chitosan mechanisms. The bactericidal properties of zinc make it a potent factor in the efficacy of antibacterial activity.

**Table 5:** The comparison of the antibacterial activity of chitosan, ZnO NPs, and chitosan–ZnO NPs in comparison to the literature data.

Sample	Zone of inhibition (mm)				Ref.
	Conc (mg/ml)	Gram-negative		Gram-positive	
		*Pseudomonas aeruginosa*	*Staphulococcus aureus*	*Listeria innocua*	
Chitosan	6	19	7	6	^75^
Chitosan	6	22	14	11	^75^
ZnO NPs	6	16	14	22	^76^
ZnO NPs	6	13	16	19	^76^
Chitosan–ZnO NPs	0.04	–	11	–	^77^
Chitosan–ZnO NPs	0.008	0	0	0	^78^

### Radical scavenger

**Figure 13:**
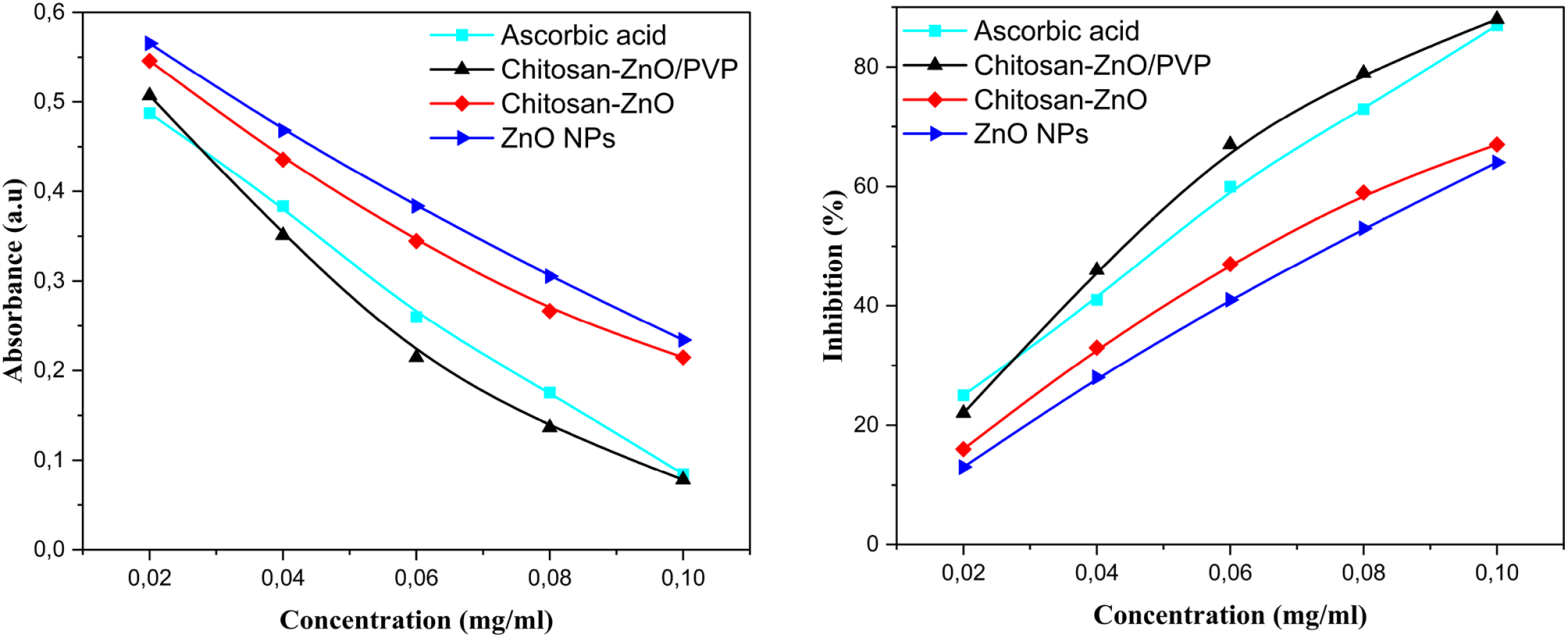
ABTS radical scavenging activity of ascorbic acid, ZnO NPs, chitosan–ZnO and chitosan–ZnO/PVP.

Figure 13 shows the results of ZnO NPs, chitosan–ZnO, and chitosan–ZnO/PVP in inhibiting ABTS free radicals, where the maximum inhibition capacities of each were 63%, 67%, and 87%. These values reveal their ability to effectively neutralize ABTS radicals. It was observed that as concentrations increased, the absorbance values decreased and the inhibition percentage increased (Fig. 13a). This indicates a relationship that depends on concentration: the higher the concentration, the greater the inhibition rate (Fig. 13b).

Comparison of the radical scavenging ability of ZnO NPs, chitosan–ZnO, and chitosan–ZnO/PVP with ascorbic acid showed that all samples exhibited significant radical scavenging ability. This result is consistent with previous studies highlighting the scavenging potential of chitosan and zinc oxide nanoparticles^80^.

**Figure 14:**
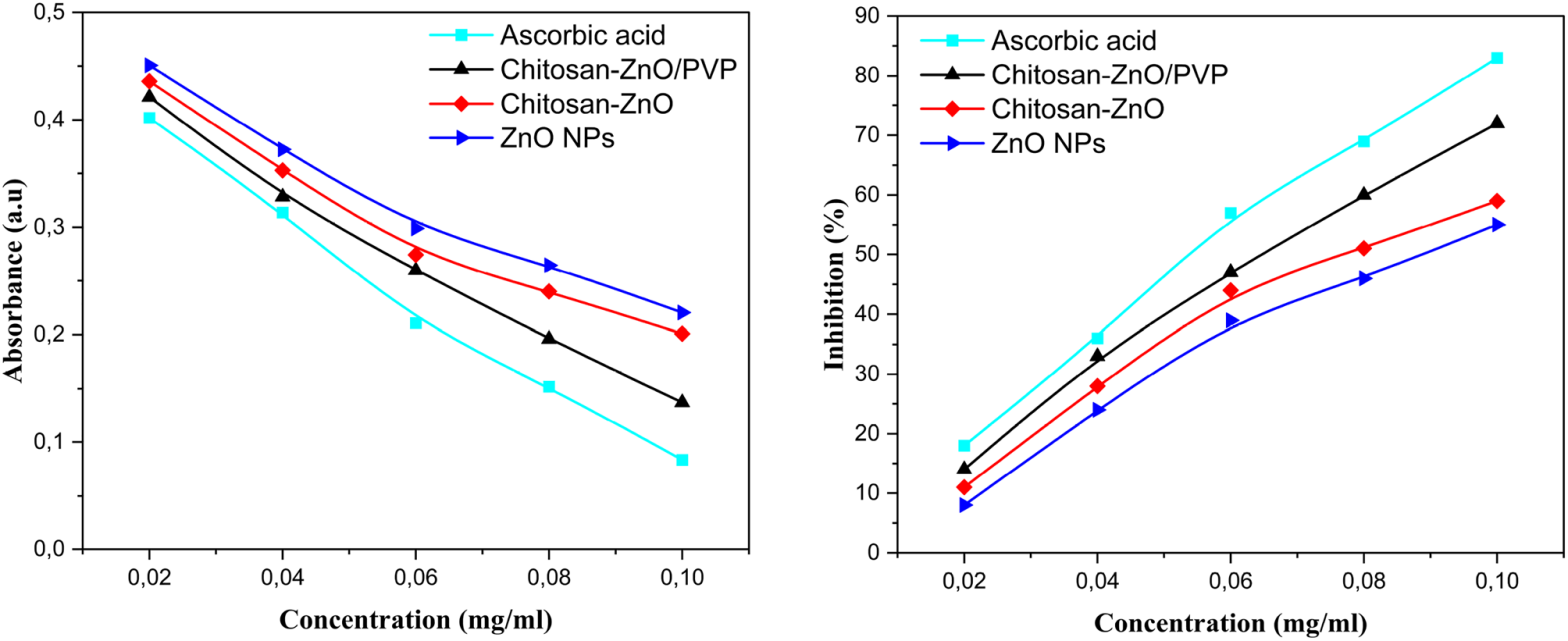
DPPH radical scavenging activity of ascorbic acid, ZnO NPs, chitosan–ZnO and chitosan–ZnO/PVP.

The free radical and DPPH antioxidant activities of ZnO NPs, chitosan–ZnO, and chitosan–ZnO/PVP at different concentrations were also examined (Fig. 14a). The maximum inhibition percentages were 51%, 56% and 72%, respectively. These values indicate their effectiveness in neutralizing DPPH radicals, it was observed that as concentrations increased, the absorbance values decreased and the inhibition percentage increased (Fig. 14b). A concentration-dependent relationship was similarly observed for DPPH, with decreased inhibition capacities at lower concentrations.

The IC_50_ results (Table 6) were as follows: 0.092, 0.079, 0.059, and 0.045 mg/mL for ZnO NPs, chitosan–ZnO, chitosan–ZnO/PVP, and ascorbic acid, respectively, in the ABTS test, and 0.095, 0.083, 0.061, and 0.064 mg/mL in the DPPH test, respectively. Incorporation of zinc into chitosan matrices and subsequently into PVP matrices significantly improved the free radical removal efficiency due to the synergistic effects of zinc, chitosan, and PVP.

**Table 6:** IC_50_ of antiaxidant activities of ZnO NPs, chitosan–ZnO and Chitosan–ZnO/PVP.

Sample	IC_50_ (mg/mL)	
	ABTS	DPPH
ZnO NPs	0.092 ± 0.09	0.095 ± 0.16
Chitosan–ZnO	0.079 ± 0.21	0.083 ± 0.39
Chitosan–ZnO/PVP	0.059 ± 0.11	0.061 ± 0.01
Ascorbi acid	0.045 ± 0.03	0.064 ± 0.04

Zinc exerts its inhibitory effect on ABTS and DPPH radicals through electron donation and redox reactions. This unique property, attributed to the high surface area and reactivity of zinc, enables zinc nanoparticles to donate electrons to ABTS and DPPH radicals, reducing them to stable, non-radical forms and preventing oxidative damage^81^. In the ABTS assay, reduction of ABTS radicals by zinc nanoparticles results in a decrease in color intensity, indicating ABTS inhibition. Likewise, scavenging of DPPH radicals reduces the intensity of the purple color of the DPPH solution, turning it yellow, indicating antioxidant activity^82^.

Chitosan shows its inhibitory effect on ABTS and DPPH radicals through its antioxidant properties and its ability to scavenge free radicals. As a biopolymer derived from chitin, chitosan is known for its antioxidant activity, possessing amino groups that effectively react with free radicals, including ABTS radicals. These amino groups (–NH2) act as electron donors, neutralizing radicals^79^. Moreover, chitosan can undergo a hydrogen atom transfer process, which enhances its scavenging ability.

Incorporating chitosan–ZnO into the PVP matrix enhances its efficiency in neutralizing free radicals by combining the antioxidant properties of chitosan and zinc oxide with the excellent dispersing and stabilizing capabilities of PVP. This integration ensures uniform distribution of active sites, improves solubility and bioavailability, and increases electrostatic interactions and hydrogen bonding with free radicals. In addition, the photocatalytic activity of zinc oxide is stabilized within the matrix, resulting in more effective and sustained free radical scavenging.

#### Predictions of organ-specific toxicity using ProTox II

**Figure 15:**
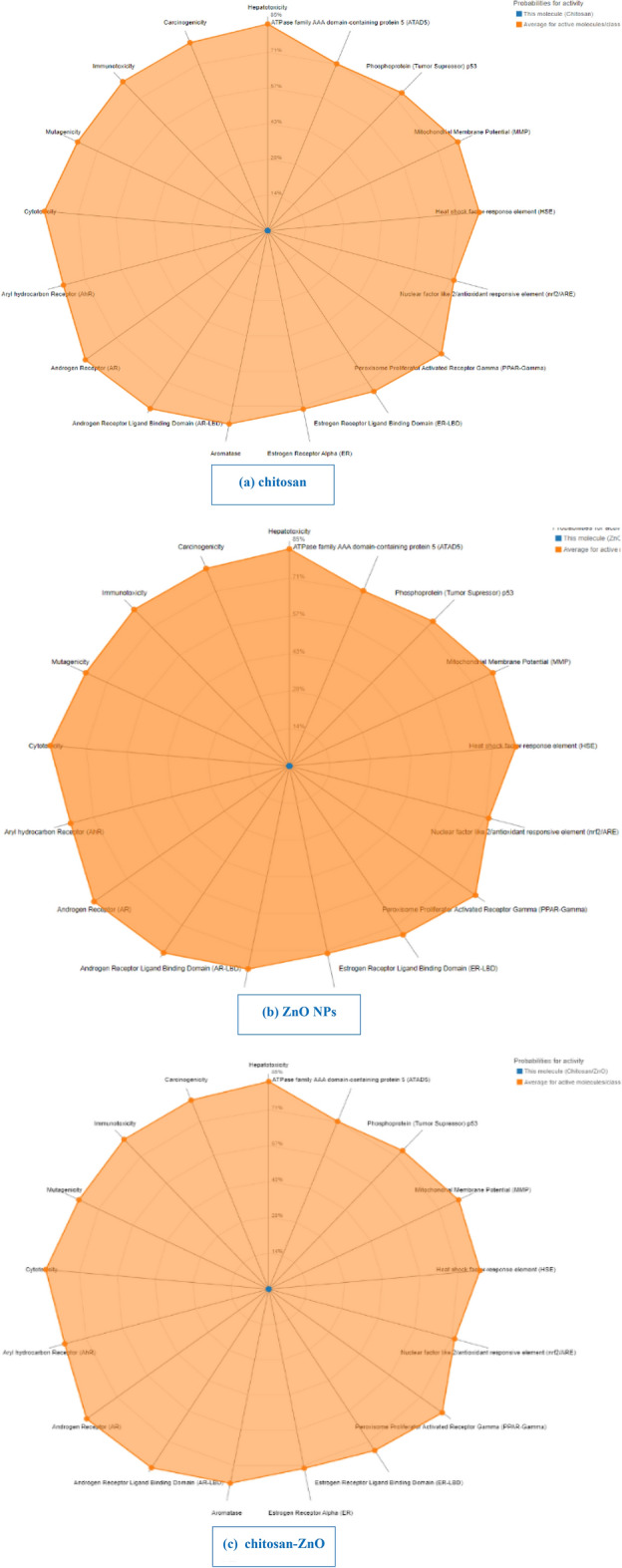
The organ-specific toxicity analysis of (**a**) chitosan; (**b**) ZnO NPs; and (**c**) chitosan–ZnO NPs.

The toxicity of the screened products was examined at three distinct levels. Some toxicity endpoints include hepatotoxicity, carcinogenicity, immunotoxicity (inhibition of the B cell development), mutagenicity, and cytotoxicity. Different toxicological pathways (Aryl hydrocarbon Receptor (AhR), Androgen Receptor (AR), Aromatase, and Estrogen Receptor Alpha (ER)). Moreover, Additionally, ATPase family AAA domain-containing protein 5 (ATAD5) and mitochondrial membrane potential (MMP)) were used to evaluate the stress response pathways. Table 7 and Fig. 15 display the forecasts’ outcomes. Based on comparison of data with relevant toxicity indicator databases, products are coded as “inactive” and “active” for “non-toxic” and “toxic” respectively. According to the toxicity evaluation, the three samples examined were found to be safe and did not have any organ-specific toxicity. According to the in silico toxicity assessment, all the examined samples are predicted to be safe and to have no organ-specific toxicity. The in silico toxicity prediction with ProTox II suffers a number of limitations. These include the uncertainity and the limited reliability of these results. Accordingly, additional in vitro and/or in vivo studies are recommended to know the real toxicity upon adminstration of the studied nanoparticles.

**Table 7:** Predictions of organ-specific toxicity using ProTox II.

Classification	Target	Chitosan		ZnO NPs		Chitosan/ZnO	
		Prediction	Probability	Prediction	Probability	Prediction	Probability
Organ toxicity	Hepatotoxicity	Inactive	0.74	Inactive	0.97	Inactive	0.97
Toxicity endpoints	Carcinogenicity	Inactive	0.69	Inactive	0.66	Inactive	0.57
	Immunotoxicity	Inactive	0.83	Inactive	0.99	Inactive	0.99
	Mutagenicity	Inactive	0.62	Inactive	0.59	Inactive	0.85
	Cytotoxicity	Inactive	0.65	Inactive	0.79	Inactive	0.78
Tox21-Nuclear receptor signaling pathways	Aryl hydrocarbon receptor (AhR)	Inactive	0.97	Inactive	0.97	Inactive	1.0
	Androgen receptor (AR)	Inactive	0.92	Inactive	1.0	Inactive	1.0
	Androgen receptor ligand binding domain (AR-LBD)	Inactive	0.96	Inactive	0.88	Inactive	0.99
	Aromatase	Inactive	0.98	Inactive	0.93	Inactive	0.82
	Estrogen receptor alpha (ER)	Inactive	0.69	Inactive	0.94	Inactive	0.99
	Estrogen receptor ligand binding domain (ER-LBD)	Inactive	0.94	Inactive	0.86	Inactive	1.0
	Peroxisome proliferator-activated receptor gamma (PPAR-Gamma)	Inactive	0.99	Inactive	0.86	Inactive	0.99
Tox21-Stress response pathways	Nuclear factor (erythroid-derived 2)-like 2/antioxidant responsive element (nrf2/ARE)	Inactive	0.98	Inactive	0.79	Inactive	0.99
	Heat shock factor response element (HSE)	Inactive	0.98	Inactive	0.79	Inactive	0.99
	Mitochondrial membrane potential (MMP)	Inactive	0.95	Inactive	0.95	Inactive	0.83
	Phosphoprotein (tumor suppressor) p53	Inactive	0.95	Inactive	0.89	Inactive	0.99
	ATPase family AAA domain-containing protein 5 (ATAD5)	Inactive	0.98	Inactive	0.88	Inactive	0.99

Based on the comparison between in vivo toxicity studies and toxicity profiles generated using the ProTox-II method (Table 8), several insights can be gleaned regarding the ability of ProTox-II to predict toxicity across different endpoints.

**Table 8:** Comparison of in vivo toxicity study results and computational prediction of toxicity profiles of chemicals using the ProTox-II method.

Toxicity in vivo			Toxicity profiles of the chemicals-using ProTox-II method							
			Carcinogenicity		Immunotoxicity		Mutagenicity		Cytotoxicity	
Compound	Toxicity	References	Prediction	Probability	Prediction	Probability	Prediction	Probability	Prediction	Probability
Isoeugenol	Strong	^85^	Active	0.55	Active	0.85	Inactive	0.97	Inactive	0.85
DNCB (positive control)	Extreme strong	^86^	Inactive	0.93	Inactive	0.65	Active	0.98	Inactive	0.73
Formaldehyde	Strong	^87^	Active	0.78	Inactive	0.99	Active	0.83	Inactive	0.82
Paraphenylenediamine	Extreme strong	^87^	Inactive	0.85	Inactive	0.99	Active	0.84	Inactive	0.75
Amylcinnamaldehyde	Moderate	^85^	Inactive	0.61	Inactive	0.80	Inactive	0.95	Inactive	0.62
Cinnamaldehyde	Moderate	^87^	Inactive	0.71	Inactive	0.98	Active	0.72	Inactive	0.92
Hydroxycitronellal	Moderate	^85^	Inactive	0.67	Inactive	0.94	Inactive	0.87	Inactive	0.86
Geraniol	Weak	^85^	Inactive	0.76	Inactive	0.99	Inactive	0.97	Inactive	0.86
Cinnamic alcohol	Weak	^85^	Inactive	0.84	Inactive	0.98	Inactive	0.94	Inactive	0.93
Eugenol	Weak	^87^	Inactive	0.73	Inactive	0.83	Inactive	0.97	Inactive	0.90
Para amino benzoic acid (PABA)	Weak	^88^	Inactive	0.62	Active	0.96	Inactive	0.97	Inactive	0.93

Based on the comparison between previous studies (Table 9), it can be concluded that toxicity prediction using ProTox-II shows results consistent with experimental results in toxicity assessment.

**Table 9:** In vivo toxicity study results in previous studies.

Compound	Toxicity	References
Chitosan NPs	Non-toxic	^89^
Chitosan NPs	Non-toxic	^90^
Chitosan NPs	Non-toxic	^91^
ZnO NPs	Non-toxic	^92^
ZnO NPs	Non-toxic	^93^
ZnO-CTS	Non-toxic	^93^
ZnO-PEG	Non-toxic	^93^

In evaluating carcinogenicity, immunotoxicity, mutagenicity, and cytotoxicity, ProTox-II demonstrates promising predictive capabilities, aligning closely with experimental findings in many cases. For instance, compounds like isoeugenol and formaldehyde exhibit strong toxicity in both in vivo studies and ProTox-II predictions, while weaker compounds such as geraniol and cinnamic alcohol show negligible toxicity in both assessments. However, there are instances where discrepancies arise, particularly with compounds like para amino benzoic acid (PABA), where ProTox-II predicts activity in immunotoxicity despite in vivo studies suggesting otherwise. Overall, while ProTox-II shows promise in predicting toxicity across various endpoints, further refinement and validation are necessary to enhance its accuracy and reliability, ultimately aiding in the early identification of potentially hazardous chemicals and guiding safer product development processes. The use of diverse molecular descriptors and machine learning algorithms, though resulting in some variability, reflects a rich and adaptable platform capable of evolving with scientific advancements. The current models’ performance metrics, such as accuracy, highlight the platform’s robust foundation, upon which further refinements can be built. Addressing species-specific and inter-individual genetic differences represents an exciting frontier for future updates, promising even more personalized and precise toxicity predictions. Additionally, the ongoing commitment to updates ensures that ProTox-II will continue to integrate the latest data and scientific insights, expanding its predictive capabilities to include new endpoints like genotoxicity, nephrotoxicity, neurotoxicity, and cardiotoxicity. Notably, several studies have successfully utilized ProTox-II, demonstrating its practical accuracy and reliability in real-world applications. This continuous evolution positions ProTox-II as a dynamic and forward-looking tool, ever-improving to meet the complex demands of drug discovery and risk assessment^83,84^.

## Conclusions

In conclusion, this study presents a green biosynthetic method of chitosan-encapsulated ZnO NPs and chitosan–ZnO/PVP with exceptional antibacterial characteristics. These NPs have also demonstrated efficient dye removal abilities on MB and TB Azo dyes. The toxicity assessment of the studied nanoparticles indicated their suitability for possible applications. Given the findings of the study, the authors recommend that it would be worthwhile exploring the scalability of the green biosynthetic strategy used for the production of chitosan–ZnO NPs. Our future studies will consider a deeper evaluation of the pharmacokinetic properties of these nanoparticles in vivo. As well, the assessment of toxicity could be evaluated in vitro and in vivo rather than in silico. Possible uses for these nanoparticles certainly go beyond their antibacterial and dye-removal capabilities, and could include medicine delivery systems or the environmental cleanup.

## Data Availability

The datasets used and/or analysed during the current study available from the corresponding author on reasonable request.
